# NIR‐II‐Trackable LYTACs Phyto‐Nanotheranostics for Source‐Microenvironment Dual‐Track ROS Regulation in Acute Gouty Arthritis

**DOI:** 10.1002/advs.75889

**Published:** 2026-05-30

**Authors:** Jian Zhang, Yingying Wang, Lujie Yu, Chunmei Jiang, Haoyu Liu, Yaohua Chen, Shutong Wu, Qin Liu, Lingling Wei, Xiaochun Zheng, Huifang Hao, Mengting Gao, Weiwei Kang, Rong Dai, Ziliang Zheng, Ruiping Zhang

**Affiliations:** ^1^ Laboratory of Molecular Imaging Fifth Hospital of Shanxi Medical University (Shanxi Provincial People's Hospital) Taiyuan China; ^2^ Shanxi Bethune Hospital, Shanxi Academy of Medical Sciences, Third Hospital of Shanxi Medical University Tongji Shanxi Hospital Taiyuan China

**Keywords:** Acute gouty arthritis, immunotherapy, macrophage, NADPH oxidase 2, TPR‐LYTACs

## Abstract

The acute gouty arthritis (GA) is critically driven by the NOX2‐mediated reactive oxygen species (ROS) explosion in macrophages, while current clinical drugs lack precise regulatory capability. Dealing with the challenge, a novel nanomaterial named TPR‐LYTACs has been developed in this study. The TPR‐LYTACs construction involves the assembly of a nano‐core (TPR) from tea polyphenols and acidified rutin. The antioxidant TPR core is conjugated via modular assembly with a NOX2‐targeting antibody and maleylated bovine serum albumin for source inhibition, enabling dual‐pathway ROS regulation. In the acute GA model rats, excellent targeting and effective control of local inflammation are demonstrated by the TPR‐LYTACs, with near‐infrared‐II fluorescence imaging confirming their specific enrichment at disease sites. Consequently, a new therapeutic strategy defined as “source inhibition and microenvironment scavenging of ROS” is established, offering a novel paradigm for the precise treatment of inflammatory diseases.

## Graphical abstract

The TPR‐LYTACs nanomaterial offers a dual‐pathway strategy for precise gouty arthritis treatment by targeting and degrading the ROS‐producing enzyme NOX2 inside macrophages while its antioxidant core clears accumulated ROS outside. This “source inhibition and microenvironment scavenging” approach, guided by NIR‐II imaging, effectively controls local inflammation.

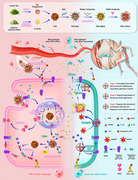

## 1 Introduction

Gouty arthritis (GA) represents a growing public health challenge worldwide, with projections indicating that nearly 95.8 million people will be affected by 2050 [1]. The disease is currently understood as a metabolic disorder characterized by hyperuricemia, which leads to local accumulation and precipitation of needle‐shaped monosodium urate crystals in the joints [2]. The GA inflammation manifests clinically as acute flares of excruciating pain, swelling, and redness in the affected joints, severely impairing patients' daily activities and diminishing their quality of life [3]. These monosodium urate (MSU) crystals are subsequently phagocytosed and recognized by macrophages, which promotes reactive oxygen species (ROS) generation and then triggers inflammasome (NLRP3, NOD‐like receptor family pyrin domain containing 3) activation and the release of numerous inflammatory cytokines, including interleukins (IL‐1β), ultimately resulting in the onset of acute GA [3, 4]. Current first‐line therapies for acute GA (colchicine, nonsteroidal anti‐inflammatory drugs [NSAIDs], and corticosteroids) are hampered by significant long‐term adverse effects, limiting their utility for recurrent or chronic use [5]. A more critical gap lies in their lack of precision: broad‐spectrum anti‐inflammatory agents fail to target the localized articular inflammatory microenvironment and scavenge ROS effectively, leaving unmet clinical needs for targeted interventions.

Accumulating evidence identifies NOX2 (NADPH oxidase 2) catalyzes NADPH to produce ROS explosion as a central driver of the acute inflammatory cascade in GA joints [6, 7]. Despite this, clinically approved therapies explicitly designed to modulate ROS homeostasis remain elusive, highlighting an urgent need for novel strategies targeting the NOX2‐ROS axis. Unfortunately, current clinical management lacks therapies designed for precise ROS regulation. The unmet need has prompted the development of novel strategies that concurrently target to achieve source inhibition and microenvironment clearance of ROS.

The activation of NOX2 in macrophages is directly induced by the deposition of MSU crystals and oxidized substances such as oxidized low‐density lipoprotein (ox‐LDL) within the joint cavity [8, 9]. Notably, gp91 phox, also known as NOX2, serves as the catalytic core subunit of the NOX2 complex, and its expression and activation are closely associated with the inflammatory status of macrophages. NOX2 is a key multi‐subunit complex on the macrophage membrane. Its activation involves the assembly of translocated cytosolic regulatory subunits (p47 phox, p67 phox, p40 phox, and RAC1‐GTPase) with the membrane‐bound catalytic subunits (gp91 phox and p22 phox) [7]. During GA progression, MSU crystal deposition and oxidative stimuli such as ox‐LDL can activate macrophages and promote the upregulation of gp91 phox expression as well as the assembly of the NOX2 complex. The activated NOX2 complex catalyzes the transfer of electrons from NADPH to O_2_. The reaction generates superoxide anions (•O_2_^−^) and then triggers an explosion of ROS, which participate in various inflammatory processes [7, 10]. Therefore, gp91 phox is not only essential for the catalytic activity of NOX2 but also represents a critical molecular link between oxidative stress and inflammatory signaling, making it an attractive therapeutic target for precise GA intervention. Although apocynin and related compounds are commonly used in research to inhibit NOX2, their practical utility is limited by unresolved issues, including off‐target toxicity and inefficient targeted delivery [11]. Furthermore, the membrane localization of NOX2 makes it difficult for conventional intervention strategies to achieve efficient regulation within the membrane microenvironment.

Recently, lysosome‐targeting chimeras (LYTACs) have been developed as a promising technology, employing membrane protein endocytosis mechanisms for precise degradation of target proteins [12]. The LYTACs technology has been successfully applied in the immunotherapy of tumors and other diseases for degrading membrane protein targets such as PD‐L1 and CD47 [13, 14]. However, the traditional LYTACs technology is constrained by the fact that its effector molecules are mostly small molecules or short peptides [15]. Owing to the instability of LYTACs molecules in the complex internal body environment, the biomacromolecules are susceptible to rapid protease degradation in the plasma and liver, which results in low bioavailability [16]. More critically, the therapeutic efficacy of LYTACs molecules is contingent upon their intact delivery to the disease site, and the requirement currently hampered by the lack of efficient and targeted in vivo delivery systems. Furthermore, due to the lack of tissue penetration of small‐molecule drugs, LYTACs are difficult to enrich in specific diseased tissues effectively. The off‐target effect not only reduces efficacy but also causes toxicity issues due to the nonspecific degradation of proteins in healthy tissues [15, 17]. To address these core challenges, nanocarrier‐based delivery systems have been proposed as a promising solution in clinical therapy [18, 19]. Nanomaterials are considered an ideal foundation for constructing such integrated platforms owing to their unique size effects and engineerable characteristics. Notably, multiple functional modules can be simultaneously loaded due to their large specific surface area, and precise targeting molecules can be assembled through surface modifiability [20, 21]. The strong permeability and retention effect inherent to nanocarriers is used to achieve passive targeting and substantial accumulation in pathological tissues, thereby significantly increasing the local concentration of therapeutic agents at the disease site. Simultaneously, the endocytic‐lysosomal transport pathway utilized by nanocarriers is naturally aligned with the operational mechanism of LYTACs. The synergistic effect of nanocarriers and LYTACs technology actively facilitates the delivery of drugs into the lysosomal compartment, and the degradation efficiency of disease‐causing proteins is enhanced.

The progression of gout is closely linked to ROS, however, the rapid burst generation in affected tissues often exceeds the regulatory capacity of LYTACs technology alone, hindering effective intervention. The optimal therapeutic strategy requires combined suppression of NOX2‐mediated ROS generation and scavenging of accumulated ROS in the microenvironment for synergistic enhancement of anti‐inflammatory effects. It is noteworthy that several natural bioactive compounds have shown considerable antioxidant potential. The curcumin directly neutralizes various free radicals via its phenolic hydroxyl structure, and resveratrol activates the Nrf2/ARE signaling pathway to strengthen cellular resistance to oxidative stress [22, 23]. In this context, the integration of phytochemical‐derived antioxidants with multifunctional nanoplatforms offers a new opportunity for disease intervention. Such a strategy can be conceptualized as phyto‐nanotheranostics, namely, a plant‐derived nanotheranostic platform that combines phytochemical‐based therapy with imaging and precision delivery functions. By integrating such natural ROS‐scavenging agents into nanocarriers, a comprehensive ‘source inhibition to microenvironment clearance’ chain functional nanoscale LYTACs strategy can be developed. The approach is expected to achieve coordinated treatment spanning molecular intervention to microenvironment remodeling, offering a new direction for the precise clinical management of gout.

**SCHEME 1:**
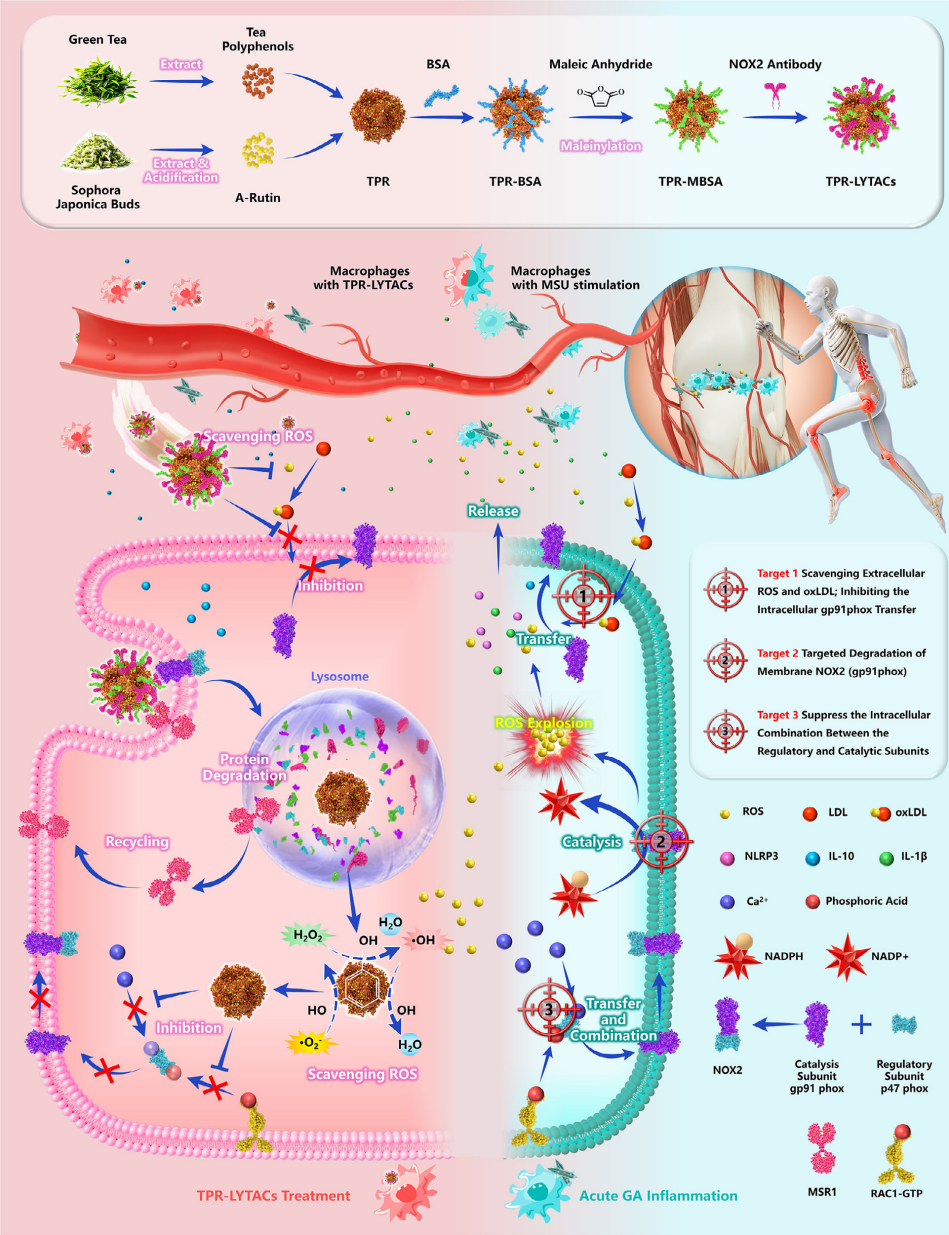
Schematic illustration of the TPR LYTACs for source‐microenvironment dual‐track ROS regulation in Acute Gouty Arthritis. (A) Schematic synthesis illustration of the TPR LYTACs. (B) Dual track ROS scavenging mechanism of TPR‐LYTACs in source‐microenvironment for acute gouty arthritis therapy.

In our study, a key objective was the scavenging of pre‐existing ROS coupled with simultaneous inhibition of the catalytic pathway responsible for ROS explosion (Scheme 1A). Guided by the concept of phyto‐nanotheranostics, two plant‐derived bioactive compounds with demonstrated antioxidant potential, tea polyphenols from green tea and rutin from the sophora japonica, were selected for the construction of a tea polyphenol‐rutin nano‐core (TPR) via molecular self‐assembly. Tea polyphenols and the flavonoid rutin share the ability to efficiently scavenge ROS through a common mechanism involving electron or hydrogen donation from their phenolic hydroxyl residues [11]. Additionally, acidified rutin (A‐Rutin) is characterized by intrinsic fluorescence in the second near‐infrared (NIR‐II) region [24]. These properties establish both compounds as ideal ROS scavengers and provide a molecular foundation for constructing the nano‐core base of LYTACs. Based on antibody‐drug conjugate technology, the TPR core was conjugated with a NOX2 (gp91 phox)‐specific antibody and maleylated bovine serum albumin (Mal‐BSA). Mal‐BSA serves as a ligand that is specifically recognized and bound by scavenger receptor type I (MSR1), highly expressed on macrophage surfaces, thereby facilitating precise intracellular delivery via the receptor's lysosome‐targeting transport mechanism. Accordingly, a TPR‐LYTACs nanomaterial was developed that integrates near‐infrared region II fluorescence (NIR‐II FL) imaging, ROS scavenging, and dual targeting of NOX2 and MSR1. The TPR‐LYTACs were designed to exert simultaneous extracellular and intracellular functions (Scheme 1B). In the extracellular microenvironment, harmful oxidative species such as oxidized low‐density lipoprotein (oxLDL) can be cleared by TPR core, thereby reducing inflammatory differentiation of macrophages. When TPR‐LYTACs simultaneously bind to both the NOX2 complex and MSR1 on macrophages, NOX2 is transported specifically to lysosomes for degradation. Meanwhile, MSR1 is recycled back to the cell membrane for reuse, enabling continuous promotion of target protein degradation. The TPR core is released into the cytoplasm following lysosomal degradation of surface proteins. Within the cytoplasm, the TPR core scavenges ROS and disrupts the assembly between the regulatory subunit p47phox and catalytic subunit gp91phox of NOX2. In summary, the dual‐tracks strategy of TPR‐LYTACs, which achieves both ROS clearance and source‐level blockade of ROS generation, represents a novel paradigm for the precise treatment of gout and inflammatory disorders.

## 2 Results and Discussion

### 2.1 Preparation and Physicochemical Characterization of TPR‐LYTACs

**FIGURE 1:**
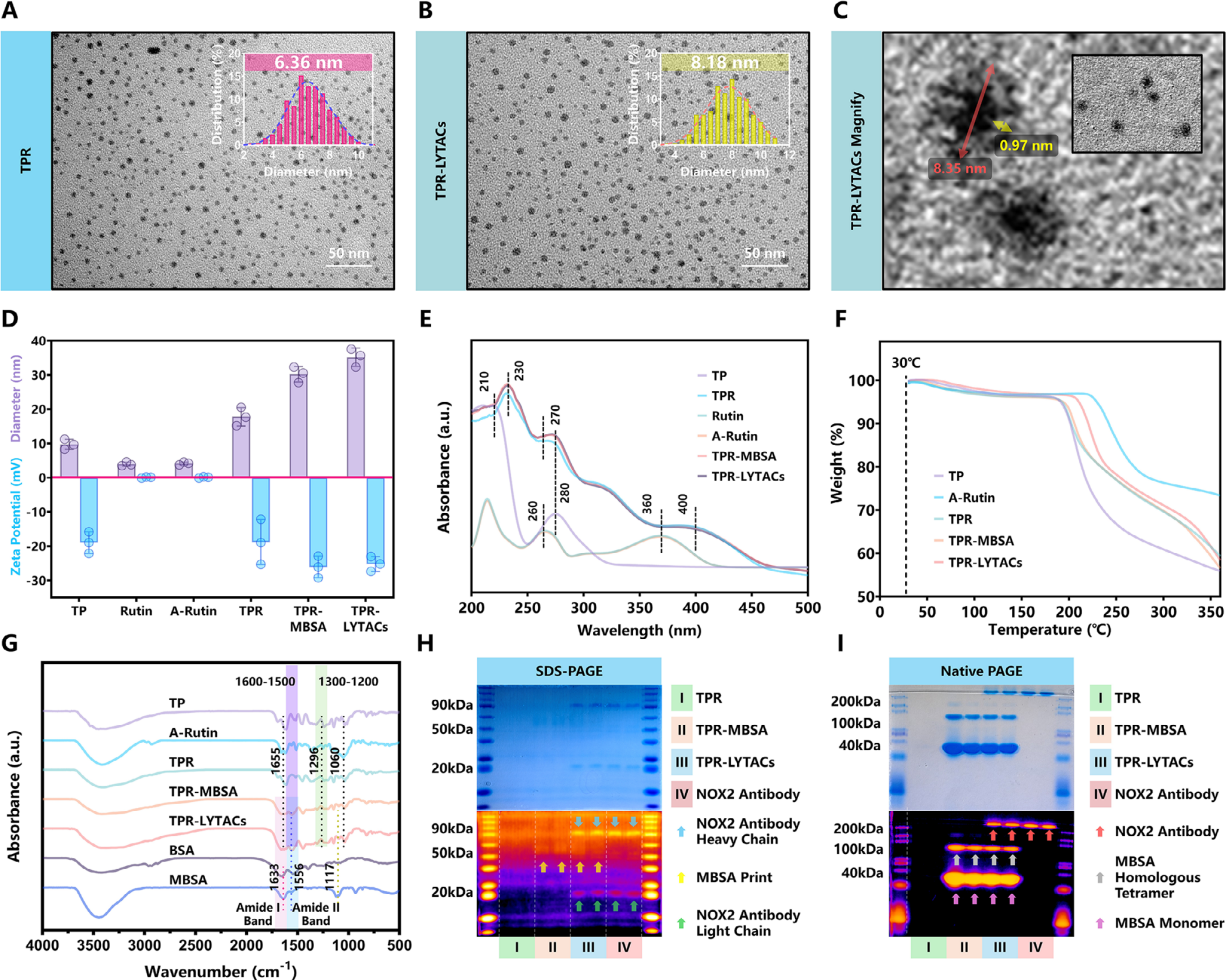
Characterization of TPR‐LYTACs. (A and B) TEM image of TPR and TPR‐LYTACs, where the size distribution is shown in the inset. (C) TEM image with different magnifications of TPR‐LYTACs. (D). DLS and zeta potentials of different raw materials and TPR relative nanomaterials. (E) The UV–vis spectra in each group of aqueous dispersion. (F) TG weight loss profiles of different raw materials and TPR relative nanomaterials. (G) FTIR spectroscopy of different raw materials and TPR relative nanomaterials. (H) SDS‐PAGE for TPR relative nanomaterials and positive group (NOX2 antibody). (I) Native PAGE for TPR relative nanomaterials and positive group (NOX2 antibody). Mean±SD, *n* = 3.

In our study, a multi‐step synthesis strategy was employed to fabricate TPR‐core nanomaterials through the self‐assembly of TP and A‐Rutin. And then TPR was followed by coating with a small amount of BSA, subsequent maleinization modification, and finally conjugated with acidified NOX2 antibody. As observed by the transmission electron microscopy (TEM) image, the synthesized TPR core nanoparticles exhibited uniform quasi‐spherical structures with relatively homogeneous size distribution, measuring approximately 6.54 ± 1.42 nm in diameter (Figure 1A). After ligand protein modification and purification, the resulting TPR‐LYTACs complexes maintained good dispersibility in TEM images, but their average diameter significantly increased to 8.18 ± 1.49 nm (Figure 1B). Significantly, high‐resolution TEM magnified images clearly revealed the typical structure of TPR‐LYTACs, with an electron‐dense dark spherical core of TPR surrounded by a uniform gray protein layer of lower electron density (Figure 1C). Measurement statistics indicated this protein layer had a thickness of approximately 0.97 nm. The overall size increase of TPR‐LYTACs from about 6 nm to about 8 nm further verified the successful construction of the complexes.

Dynamic light scattering (DLS) and Zeta potential analysis were systematically performed on various components during synthesis to evaluate further the successful assembly and colloidal stability of TPR‐LYTACs (Figure 1D). The results clearly demonstrated the evolution of size and surface charge from the TP and A‐Rutin to the final TPR‐LYTACs complexes. Analysis of the TP showed a hydrodynamic diameter of 9.72 ± 1.46 nm and a negative surface charge (−18.95 ± 3.16 mV), consistent with the partial deprotonation of its polyphenol hydroxyl groups in the aqueous phase. The rutin and A‐Rutin indicated both exhibited electrical neutrality in aqueous solution, primarily due to their unique molecular structures and dissociation states under measurement conditions. Under the near‐neutral or pH < 5 aqueous buffer conditions, most phenolic hydroxyl groups remained protonated and could not contribute a significant negative charge. The limited ether oxygen atoms on sugar rings underwent slight protonation (positive charge regions) and weakly dissociated phenolate anions (negative charge regions) counteracted each other in macroscopic measurements, ultimately showing a net charge close to zero [25]. Comparative analysis with TPR core's DLS diameter (17.79 ± 2.67 nm) revealed that TPR‐MBSA and TPR‐LYTACs significantly increased to 30.21 ± 2.28 nm and 35.18 ± 2.67 nm, respectively. Notably, both TPR and TPR‐LYTACs exhibited excellent dispersibility in various media, including water, PBS, FBS, DMEM, and DMEM supplemented with FBS. Even after 7 days of incubation, they remained highly stable, with negligible changes in morphology and particle size (Figure S1A,B). In addition, their polydispersity index (PDI) was monitored over the 7‐day period, and the PDI values of them consistently maintained within the range of 0.1–0.3, indicating good dispersion uniformity (Figure S1C,D). Therefore, TPR and TPR‐LYTACs possess high physiological stability and prolonged blood circulation potential, providing a solid foundation for their subsequent applications in cellular and in vivo studies. The marked size change provided direct evidence for the successful coating of high molecular weight target proteins onto the nanoparticle surfaces.

Notably, the TPR inherited the negative Zeta potential of TP (approximately −18.78 ± 6.59 mV), confirming the stability of its surface chemical environment. TPR‐MBSA introduced negatively charged sulfonic acid groups, leading to a significantly increased negative Zeta potential (approximately −26.08 ± 3.17 mV). It should be noted that during TPR‐LYTACs synthesis, acidified NOX2 antibody was introduced to impart a positive charge to the antibody Fc region. Since both Fc and Fab segments of the antibody carried positive charges, the connection with TPR‐MBSA offset part of the negative charge [26]. Consequently, TPR‐LYTACs exhibited a Zeta potential of approximately −25.20 ± 2.17 mV. The moderate negative charge carried by the final product helps enhance its colloidal stability in the aqueous phase, establishing a foundation for subsequent biological function studies.

Ultraviolet‐visible spectroscopy (UV–vis) was employed to verify the successful chemical reaction between TP and A‐Rutin. As shown in Figure 1E, the spectral characteristics of the synthetic product TPR demonstrated significant and indicative changes compared to the monomers before reaction. The red shift of the characteristic absorption peak from 210 nm in TP to 230 nm in TPR was observed. The substantial displacement of 20 nm directly indicated an expansion of the intramolecular conjugation system and enhanced electron cloud delocalization, likely originating from the formation of new covalent bonds between TP and rutin that constructed the π‐conjugation system with lower energy [27]. The UV absorption peaks of Rutin and A‐Rutin almost completely overlap. The characteristic peak of A‐Rutin at 260 nm and the characteristic peak of TP at 280 nm merged into a single broad peak at 270 nm in TPR. More notably, the characteristic absorption peak of A‐Rutin at 360 nm was further red‐shifted to 400 nm in TPR. The significant red shift reaching the edge of the visible light region strongly suggested the possible formation of a stronger intramolecular charge transfer effect within TPR, where the excited state was greatly stabilized by the expanded conjugate structure and donor–acceptor interactions [28]. Given that proteins exhibit strong absorption at 280 nm, this property was utilized to evaluate TPR‐MBSA and TPR‐LYTACs. A significant enhancement of the absorption signal at 280 nm was observed when TPR was conjugated with MBSA and NOX2 antibody.

The relationship between material structure and thermal stability of TPR‐LYTACs was further investigated through the comprehensive analysis of thermogravimetric (TG) and UV–vis results (Figure 1F). The TG curves showed an onset decomposition temperature of 188°C for TPR, between its precursors TP (186°C) and A‐Rutin (222°C). The intermediate thermal stability indicated the formation of a new compound with distinct thermodynamic properties rather than a simple mixture. The main thermal decomposition stage revealed similar degradation pathways for TPR and its protein‐modified derivatives, with curve intersections suggesting shared thermal cleavage mechanisms. Although TP and A‐Rutin demonstrated similar char residue rates after 500°C, reflecting their aromatic frameworks' tendency to form stable carbon structures at high temperatures, systematic changes in thermal decomposition behavior were observed with progressive structural modifications [29]. TPR‐LYTACs exhibited the lowest final char residue rate of 6.44% at 675°C, closely related to the molecular conjugation revealed by ultraviolet spectroscopy.

Fourier transform infrared (FTIR) spectroscopy analysis confirmed the successful formation of the TPR core nanomaterial through the reaction between TP and A‐Rutin (Figure 1G). The infrared spectrum of TPR contained characteristic absorption peaks from both TP and A‐Rutin. A strong absorption peak at 1655 cm^−1^, together with the glycosidic bond vibration at 1060 cm^−1^, indicated the presence of the A‐Rutin structure [30]. Multiple aromatic C═C stretching vibrations observed between 1600 and 1500 cm^−1^, along with C─O composite vibration peaks in the 1300–1200 cm^−1^ range, were attributed to the complex structural features of the tea polyphenol mixture [31]. These spectral characteristics collectively support that TPR is a composite formed by the chemical reaction between TP and A‐Rutin. During maleinylation modification of BSA, the amide I band of maleylated bovine serum albumin was shifted from 1656 cm^−1^ in native BSA to 1633 cm^−1^ [32]. The shift may be caused by a transition in protein secondary structure toward β‐sheet conformation, or by the overlap between the newly introduced maleimide C═O stretching vibration and the amide I band. The movement of the amide II band from 1540 cm^−1^ to 1556 cm^−1^ is regarded as a change in vibration mode resulting from the acylation of lysine amino groups to form new amide bonds, serving as reliable evidence of successful amino modification. The enhanced absorption peaks near 1117 cm^−1^ were considered related to the maleoyl group or originated from complex molecular vibrations in the fingerprint region [33]. The finally prepared TPR‐LYTACs material exhibited characteristic absorption peaks of both TPR and the protein in the infrared spectrum, providing a material basis for subsequent research on bio‐targeting applications.

The synthesized TPR and its protein complexes were characterized by sodium dodecyl sulfate polyacrylamide gel electrophoresis (SDS‐PAGE) and native PAGE (Figure 1H,I). Under denaturing conditions, no protein bands were observed in the TPR group, confirming sample purity and excluding protein contamination. Both TPR‐MBSA and TPR‐LYTACs groups showed characteristic bands of MBSA at approximately 50 kDa, verifying the presence of the MBSA core component. Crucially, the TPR‐LYTACs group displayed two bands at about 20 kDa and 90 kDa corresponding perfectly to the light and heavy chains of the NOX2 antibody, respectively. It provided direct evidence for the successful conjugation of antibody components into the TPR‐LYTACs complex. To further analyze the complete structure and assembly state of the complexes, native PAGE was performed (Figure 1I). Similarly, no protein bands were detected in the TPR group. For TPR‐MBSA, two bands were observed, one band at about 40 kDa corresponding to the monomer and another band at about 140 kDa likely originating from the inherent homologous tetramer structure of MBSA. The phenomenon indicated that TPR modification did not disrupt the inherent oligomerization capability of MBSA. Most importantly, the TPR‐LYTACs group displayed a clear band in the high molecular weight region around 200 kDa, consistent with the expected size of an intact IgG antibody. The SDS‐PAGE and native PAGE demonstrated that TPR‐LYTACs were a functionally assembled entity with correct higher‐order structure.

### 2.2 Multifunctional Evaluation of TPR‐LYTACs

**FIGURE 2:**
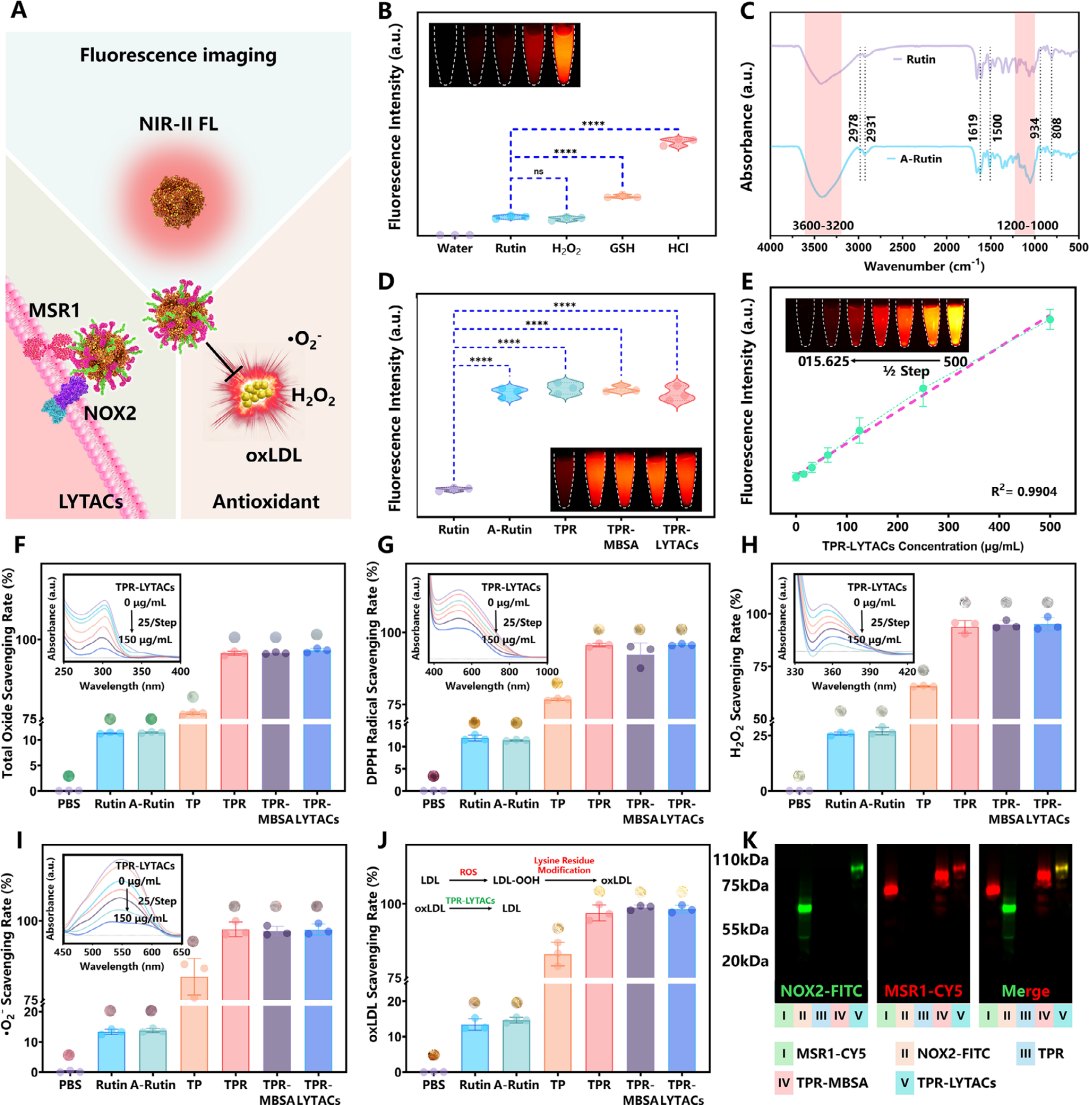
Multifunctional Evaluation of TPR‐LYTACs. (A) Multifunctional diagrammatic sketch of TPR‐LYTACs. (B) The NIR‐II FL of rutin with different chemical modification methods, including H_2_O_2_, GSH, and HCl acidification. (C) FTIR spectroscopy analysis of rutin and A‐Rutin. (D0 The NIR‐II FL of rutin, A‐Rutin, and TPR relative nanomaterials. (E) The corresponding quantitative concentration‐dependent NIR‐II FL signal values of TPR‐LYTACs. (F) Total oxide scavenging rate and UV–vis absorption of ABTs with different TPR‐LYTACs’ concentration, from 0 to 150 µg/mL, 25 µg/mL per step. (G) DPPH scavenging rate and UV–vis absorption of DPPH with different TPR‐LYTACs’ concentration (same above). (H) H_2_O_2_ scavenging rate and UV–vis absorption of H_2_O_2_ with different TPR‐LYTACs’ concentration (same above). (I) •O_2_^−^ scavenging rate and UV–vis absorption of •O_2_^−^ with different TPR‐LYTACs’ concentration (same above). (J) oxLDL scavenging rate. (K) The target binding experiments of TPR‐LYTACs by native PAGE. Mean±SD, *n* = 3, ^*^ *p *< 0.05, ^**^ *p *< 0.01, ^***^ *p *< 0.001, ^****^ *p *< 0.0001.

The multifunctional properties of the TPR material, encompassing NIR‐II FL imaging, antioxidant, and targeted binding to MSR1 and NOX2 capabilities, were systematically evaluated in this study (Figure 2A). Rutin was first treated with different chemical modification methods, including H_2_O_2_, GSH, and HCl acidification (Figure 2B). NIR‐II FL analysis showed that A‐Rutin prepared through HCl acidification exhibited stronger fluorescence emission than others in the NIR‐II region. The observation led to further investigation of its structural nature by FTIR spectroscopy (Figure 2C). In the hydroxyl stretching region, the broad, strong absorption band at 3300 cm^−1^ observed in rutin was attributed to hydrogen bonds formed by phenolic hydroxyl groups [34]. The band showed significant changes in A‐Rutin, indicating protonation of phenolic hydroxyl groups through acidification. The protonation process reduced intermolecular hydrogen bonding while enhancing hydrophobicity. Consequently, intramolecular rotation and vibrational relaxation were restricted, leading to effective suppression of the twisted intramolecular charge transfer process and decreased nonradiative relaxation. The C─H and aromatic ring vibration regions demonstrated enhanced C─H stretching vibrations at 2978 cm^−1^ and 2931 cm^−1^ in A‐Rutin [35, 36]. Simultaneously, shifts in C═C stretching vibrations were observed along with a new absorption peak at 1619 cm^−1^ [37]. The peak was assigned to coupled vibrations of C═O and C═C in the lactone structure. These spectral changes confirmed lactone formation, which enhanced molecular rigidity and induced a decrease in π–π* energy levels, accompanied by a spectral red shift. These structural modifications further suppressed nonradiative relaxation. In the 1200–1000 cm^−1^ [30] region, the strong absorption of glycosidic bond C─O stretching vibrations is associated with increased conformational rigidity, thereby contributing to enhanced fluorescence intensity. In the low wavenumber region, vibrational mode coupling near 1500 cm^−1^ was redistributed in A‐Rutin. The out‐of‐plane C─C bending vibration at 934 cm^−1^ was intensified, while the in‐plane C─C bending vibration at 808 cm^−1^ was weakened [38]. These alterations indicate improved aromatic ring planarity and reduced vibrational coupling. Internal conversion and other vibrational relaxation pathways were collectively suppressed, promoting preferential release of excited‐state energy through radiation. The fluorescence enhancement of A‐Rutin was therefore attributed to acidification‐induced hydrogen bond environment modification, rigidity improvement from lactonization, and redistribution of low‐frequency vibrational modes. These structural changes cooperatively inhibited multiple nonradiative relaxation channels, ultimately leading to significantly improved fluorescence performance. A linear response was maintained across a wide concentration range, demonstrating stable fluorescence characteristics of TPR in the NIR‐II region (Figure 2D). The fluorescence retention capability of TPR during functionalization was further verified by comparing fluorescence intensities of A‐Rutin, TPR, and its two functional derivatives, TPR‐MBSA and TPR‐LYTACs (Figure 2E). The critical finding indicated that the core fluorophore of TPR maintained structural integrity during modification processes and established a foundation for its applications in biomedical imaging and real‐time tracking in drug delivery systems.

The antioxidant capacity of TP and rutin, primarily mediated by its unique phenolic hydroxyl groups, involves distinct mechanisms for scavenging different reactive oxygen species. H_2_O_2_ was scavenged through direct reduction by phenolic hydroxyl groups, being converted to H_2_O and O_2_. Free radicals were scavenged via H^+^ transfer from phenolic hydroxyls, resulting in stable phenoxyl radicals. •O_2_^−^ was scavenged through an electron transfer pathway that converts it to O_2_ and H_2_O_2_, which underwent subsequent reduction. Based on these mechanisms, the antioxidant properties of TPR were systematically evaluated (Figure 2F–I). The TPR demonstrated significantly enhanced scavenging capacity for the above ROS subtype, reaching the combined capacity of its precursors, TP and rutin. The synergistic effect indicated the formation of a novel molecular structure with higher antioxidant activity during synthesis. Further comparison revealed no significant differences in scavenging capacity among TPR, TPR‐MBSA, and TPR‐LYTACs, suggesting the preservation of core antioxidant active sites during subsequent modifications. Meanwhile, concentration‐dependent antioxidant efficacy was observed in TPR‐LYTACs across the tested concentration ranges with continuous enhancement as concentration increased. Consistent results were obtained in oxLDL clearance assays, demonstrating the broad and effective antioxidant activity of the TPR series material (Figure 2J).

The targeted binding ability of TPR‐LYTACs to specific proteins was evaluated through binding experiments using FITC‐labeled NOX2 protein and CY5‐labeled MSR1 protein under native PAGE (Figure 2K). A green fluorescent band was observed for NOX2‐FITC at 80 kDa. A red fluorescent band was observed for MSR1‐CY5 at 90 kDa. These band positions were higher than the theoretical molecular weights of 55 kDa for NOX2 and 75 kDa for MSR1. The phenomenon may be related to the electrophoretic mobility of proteins in their native conformation. The base TPR material showed no binding to any tested proteins, demonstrating an absence of specific targeting function. TPR‐MBSA modified with MBSA formed a complex with MSR1‐CY5 at 110 kDa, and red fluorescence was detected. The molecular weight increase confirmed successful ligand‐receptor binding. TPR‐LYTACs modified with LYTACs simultaneously exhibited both red and green fluorescence at 200 kDa. The band position was significantly higher than the individual protein molecular weights. It clearly indicated the formation of a stable high‐molecular‐weight complex with both NOX2‐FITC and MSR1‐CY5 proteins, and demonstrates the outward radial directionality of the Fab region of the NOX2 antibody. These results systematically verified that different modification strategies impart distinct targeting properties to TPR derivatives. TPR‐LYTACs displayed a unique dual‐targeting capability. It could simultaneously recognize and bind two different protein targets. The property offers significant application value in targeted therapy and delivery systems that require multi‐target synergy. An experimental basis was provided for further in vitro studies. The property offers substantial application value in targeted therapy and delivery systems requiring multi‐target synergy, thus providing an experimental basis for further in vitro studies.

### 2.3 NOX2 Mediates Macrophage ROS Explosion in Gouty Arthritis

**FIGURE 3:**
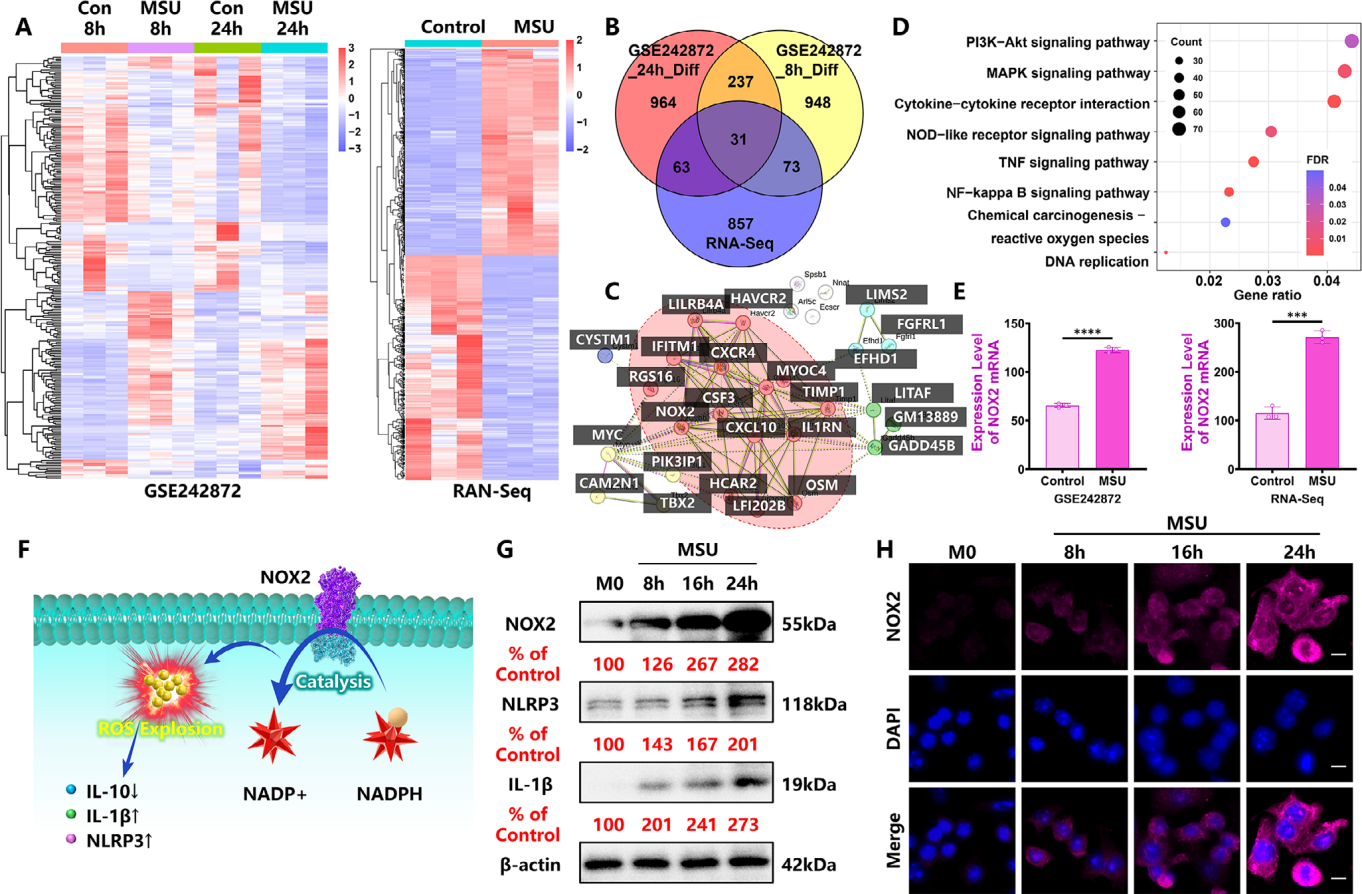
NOX2 Mediates Macrophage ROS Explosion in Gouty Arthritis. (A) The heatmap of DEGs from the control and MSU groups in both GSE242872 and our RNA‐sequencing data. (B) The Venn diagram of DEGs from the GSE242872_24 h, GSE242872_8 h, and our RNA‐sequencing data. (C) The PPI network diagram of DEGs. (D) KEGG Bubble Chart. (E) Expression level of NOX2 mRNA in both GSE242872 and our RNA‐sequencing data. (F) Schematic illustration of NOX2 catalyzing NADPH to generate ROS explosion. (G) The level of NOX2, NLRP3, and IL‐1β protein expression in different time nodes of MSU intervention was assessed by Western blot and analysis. (H) The level of NOX2 protein expression in different time nodes of MSU intervention was assessed by immunofluorescence. Scale bar 10 µm. Mean±SD, *n* = 3, ^***^ *p *< 0.001, ^****^ *p *< 0.0001.

The study integrated the transcriptomic dataset GSE242872 related to GA from the Gene Expression Omnibus database (GEO) with our RNA sequencing data from MSU‐induced macrophages (Figure 3A). A total of 2316 differentially expressed genes were identified between the MSU and control groups in the GSE242872 dataset. Of these, 1289 were specific to the 8 h MSU intervention (MSU8h group), 1295 to the 24 h intervention (MSU24h group), and 268 were common to both time points. Meanwhile, 1024 differentially expressed genes (DEGs) were detected in our RNA sequencing data of macrophages (RAW 264.7, #STCC20020P, Wuhan Servicebio Technology, Wuhan, China), comprising 491 upregulated and 533 downregulated genes. Differential gene expression heatmaps revealed numerous significantly dysregulated genes and clustering relationships across all datasets, clearly distinguishing the upregulated from downregulated genes (Figure 3A,B). To facilitate subsequent functional investigations and pathway analysis, the Venn diagram analysis was employed to identify 31 common DEGs (Figure 3B). These genes form a cross‐validated, high‐confidence core gene set that was likely to play a critical role in the MSU‐induced inflammatory response. Subsequently, the protein‐protein interaction (PPI) network was utilized to investigate the interactions among these commonly DEGs. Based on topological features, 20 highly interconnected proteins located at the network core were identified, suggesting their essential hub roles within functional modules (Figure 3C). To further elucidate the biological functions of these hub genes, KEGG pathway enrichment analysis revealed that they were significantly enriched in pathways including PI3K‐AKT, MAPK, cytokine‐cytokine receptor interaction, and NOD‐like receptor signaling, indicating their critical involvement in the pathogenesis of GA (False discovery rate, FDR < 0.05, Figure 3D). Notably, NOX2, encoded by the CYBB gene, was identified as a key molecule between the protein interaction network and NOD‐like receptor signaling pathway (Figure 3C,D). In both transcriptomic datasets, consistent upregulation of NOX2 expression was observed in MSU‐induced macrophages, further supporting its likely important role in this process (*p *< 0.001, Figure 3E).

Previous studies had established NOX2 as a key enzyme for ROS explosion in phagocytes, fulfilling its role in innate immunity and inflammation by catalyzing the oxidation of intracellular NADPH to generate an explosion of ROS [39]. Elevated ROS levels mediate oxidative stress and activate the NLRP3 inflammasome, thereby promoting the release of pro‐inflammatory cytokines (IL‐1β) and suppressing anti‐inflammatory factors (IL‐10), which amplifies the inflammatory response (Figure 3F) [40]. The above findings were further validated, and MSU‐induced macrophages were examined at different time points (Figure 3G). The NOX2 upregulation was accompanied by elevated expression of NLRP3 and IL‐1β and decreased expression of IL‐10, with all indicators exhibiting a clear time‐dependent response to MSU stimulation. Enhanced NOX2 protein expression following MSU stimulation was further confirmed by immunofluorescence assays, which also showed increasing localization signals on the cell membrane (Figure 3H and Figure S2). These findings are consistent with existing research and transcriptomic analysis results, which further deepen the understanding of the molecular mechanisms underlying gout pathogenesis and provide a theoretical basis for NOX2‐targeted therapeutic strategies.

### 2.4 TPR‐LYTACs Alleviate MSU‐Induced Inflammation

**FIGURE 4:**
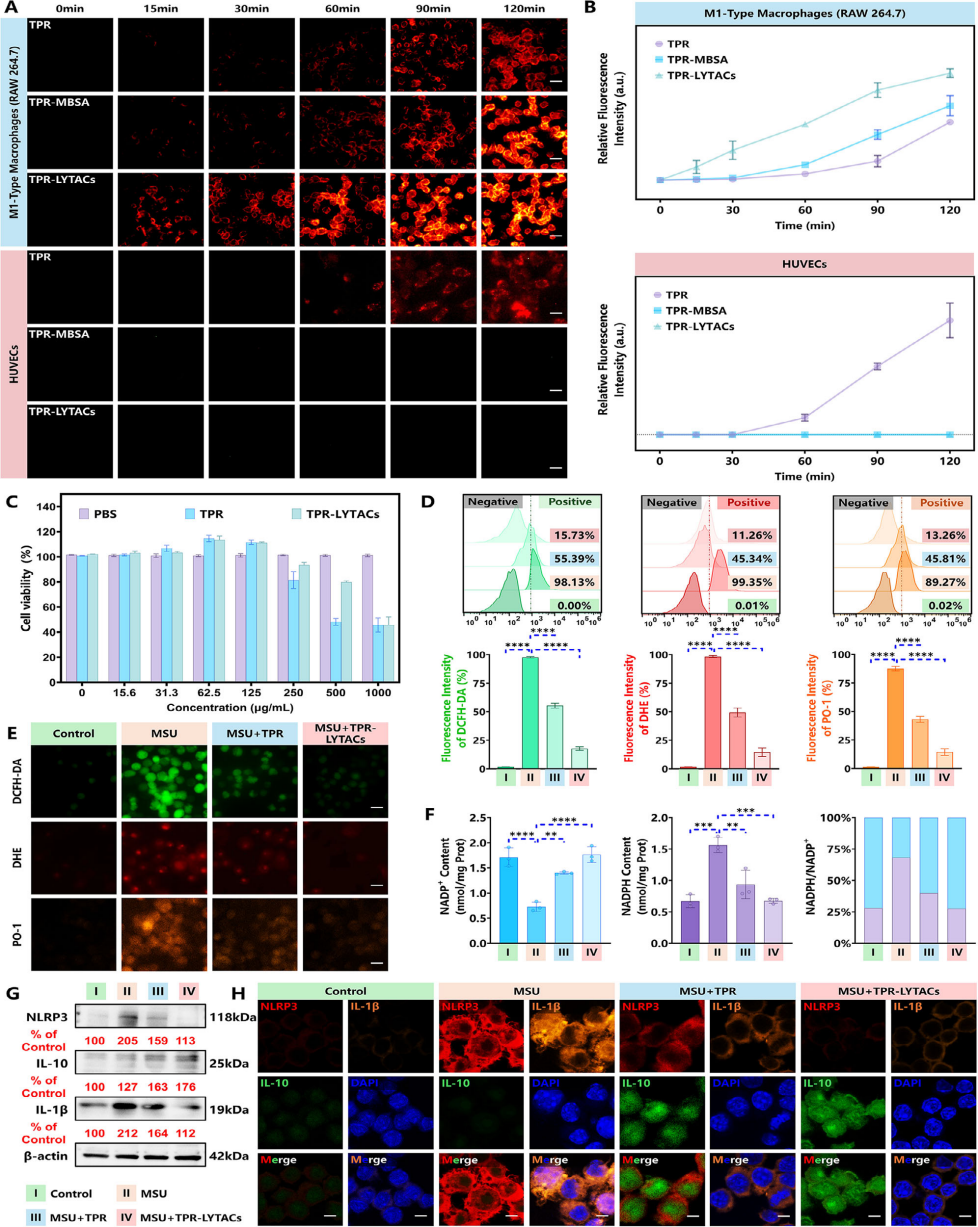
TPR‐LYTACs Alleviate MSU‐Induced ROS Explosion and Inflammation. (A and B) Microscopic NIR‐II FL images and analysis of M1‐type macrophage (RAW 264.7) and HUVECs treated with TPR, TPR‐MBSA, and TPR‐LYTACs for 0, 15, 30, 60, 90, and 120 min. Scale bar 50 µm. (C) Cell viability of M1‐type macrophage treated with TPR and TPR‐LYTACs at different concentrations for 24 h. (D) Flow cytometry and analysis of DCFH‐DA, DHE, and PO‐1 in each group. (E) The fluorescence images of DCFH‐DA, DHE, and PO‐1 in each group. Scale bar 100 µm. (F) The NADP^+^ and NADPH assay in each group. (G) The level of NLRP3, IL‐10, and IL‐1β protein expression in each group was assessed by Western blot and analysis. (H) The level of NLRP3, IL‐10, and IL‐1β protein expression in each group was assessed by immunofluorescence. Scale bar 10 µm. DAPI Exposure time 10 ms. IL‐10 Exposure time 60 ms. IL‐1β Exposure time 30 ms. NLRP3 exposure time 40 ms. Mean±SD, *n* = 3, ^*^ *p *< 0.05, ^**^ *p *< 0.01, ^***^ *p *< 0.001, ^****^ *p *< 0.0001.

A series of experiments was conducted to further evaluate the potential of the TPR‐LYTACs in alleviating MSU‐induced macrophage inflammation. The uptake kinetics of different TPR‐based nanomaterials in M1‐type macrophages were monitored in real time using NIR‐II FL imaging (Figure 4A). The unmodified TPR began to be taken up after 60 min of co‐incubation with cells, and uptake by all cells was completed by 120 min. In contrast, TPR‐MBSA showed significantly enhanced uptake speed due to improved targeting ability towards the MSR1 receptor. NIR‐II FL signals were detected as early as 30 min, complete cellular uptake was achieved by 90 min, and the peak uptake was reached at 120 min. Notably, TPR‐LYTACs exhibited very rapid uptake, with signals detected at 15 min, complete uptake achieved by 30 min, and a plateau reached at 90 min. The control experiment was performed using human umbilical vein endothelial cells (HUVECs, #STCC12103P, Wuhan Servicebio Technology, Wuhan, China) to further verify the targeting specificity of TPR‐LYTACs towards M1 macrophages (Figure 4B). The TPR began to be taken up by HUVECs after 90 min, while no significant uptake of TPR‐MBSA or TPR‐LYTACs was observed within 120 min. Delightfully, these results indicated that the dual‐ligand design of TPR‐LYTACs significantly enhanced recognition and internalization by macrophages, resulting in greatly improved uptake kinetics. The specificity of this targeting was confirmed by the absence of significant uptake in HUVECs, which lack the corresponding receptors.

Encouraged by the rapid uptake of TPR‐LYTACs in macrophages, we performed CCK‐8 assays to evaluate the cytotoxicity of TPR‐based nanomaterials at various concentrations on both HUVECs and macrophages (Figure 4C). Compared with the PBS group, TPR and TPR‐LYTACs all promoted macrophage proliferation at concentrations below 125 µg/mL. However, when their concentration exceeded 125 µg/mL, cell viability gradually declined. Since 125 µg/mL represented the highest concentration that maintained cell viability without inducing cytotoxicity, the level was selected for subsequent experiments. As seen in Figure S3A, the decrease in cell viability was caused by TPR at concentrations above 500 µg/mL in HUVECs, whereas TPR‐LYTACs showed almost no adverse effects. The phenomenon was likely due to the inability of HUVECs to efficiently uptake the modified TPR‐based nanomaterials. Overall, the findings demonstrated that TPR‐LYTACs exhibited a favorable safety profile with minimal cytotoxicity at the biologically effective concentration used in cellular experiments.

ROS explosion was recognized as the core mechanism driving M1 macrophage polarization and the release of pro‐inflammatory factors. For a comprehensive assessment of their antioxidant effects at the cellular level, intracellular total ROS levels were measured using the DCFH‐DA probe, and •O_2_^−^ and H_2_O_2_ were detected explicitly with the DHE and PO‐1 probes, respectively. As shown in Figure 4D, flow cytometric analysis revealed a distinct rightward shift in the DCFH‐DA fluorescence intensity histogram in the MSU group compared to the Control group, indicating a significant increase in intracellular ROS levels (*p *< 0.0001). Following TPR treatment, the fluorescence peak shifted noticeably to the left, with a reduction in ROS levels of approximately 50%. In the TPR‐LYTACs group, ROS levels were further decreased by about 90% (*p *< 0.0001). In addition to the intrinsic ROS clearance capability of the TPR core structure, TPR‐LYTACs may enhance intracellular ROS clearance through a LYTAC‐mediated targeted degradation mechanism. TPR‐LYTACs exhibited superior clearance capacity across all ROS categories compared to TPR alone, indicating broad‐spectrum and synergistic antioxidant functionality. Supporting these observations, cellular ROS fluorescence imaging revealed a markedly weakened fluorescence intensity in cells after TPR‐LYTACs treatment, further confirming efficient ROS clearance at the cellular level (Figure 4E). Given that ROS explosion in macrophages is highly dependent on NADPH availability, intracellular NADPH levels were further examined (Figure 4F). Compared to the MSU group, both TPR and TPR‐LYTACs treatments significantly reduced NADPH content (*p *< 0.001). The result implied that, beyond direct ROS clearance, both materials may exert antioxidant effects at an upstream level by modulating NADPH metabolism and indirectly suppressing NOX2‐mediated NADPH catalytic reaction.

Based on the notable ROS clearance effects described above, the regulatory impact of TPR‐LYTACs on key inflammatory cytokines in M1 macrophages was further investigated. Western blot and immunofluorescence staining analyses showed that TPR‐LYTACs treatment significantly suppressed NLRP3 inflammasome activation, as well as the maturation and release of IL‐1β, while promoting the expression of the anti‐inflammatory cytokine IL‐10 (Figure 4G). Grayscale analysis of protein bands indicated that TPR‐LYTACs reduced NLRP3 and mature IL‐1β expression levels by approximately 50% in MSU‐stimulated macrophages, whereas IL‐10 expression was upregulated more than 1.5‐fold. Consistent with these data, immunofluorescence images showed a significant reduction in NLRP3 and IL‐1β fluorescence intensity, an increase in IL‐10, along with a shift in cell morphology toward an anti‐inflammatory phenotype, following TPR‐LYTACs treatment (Figure 4H and Figure S3B).

### 2.5 TPR‐LYTACs Inhibit gp91 Phox Membrane Translocation by Scavenging Extracellular Ox‐LDL

**FIGURE 5:**
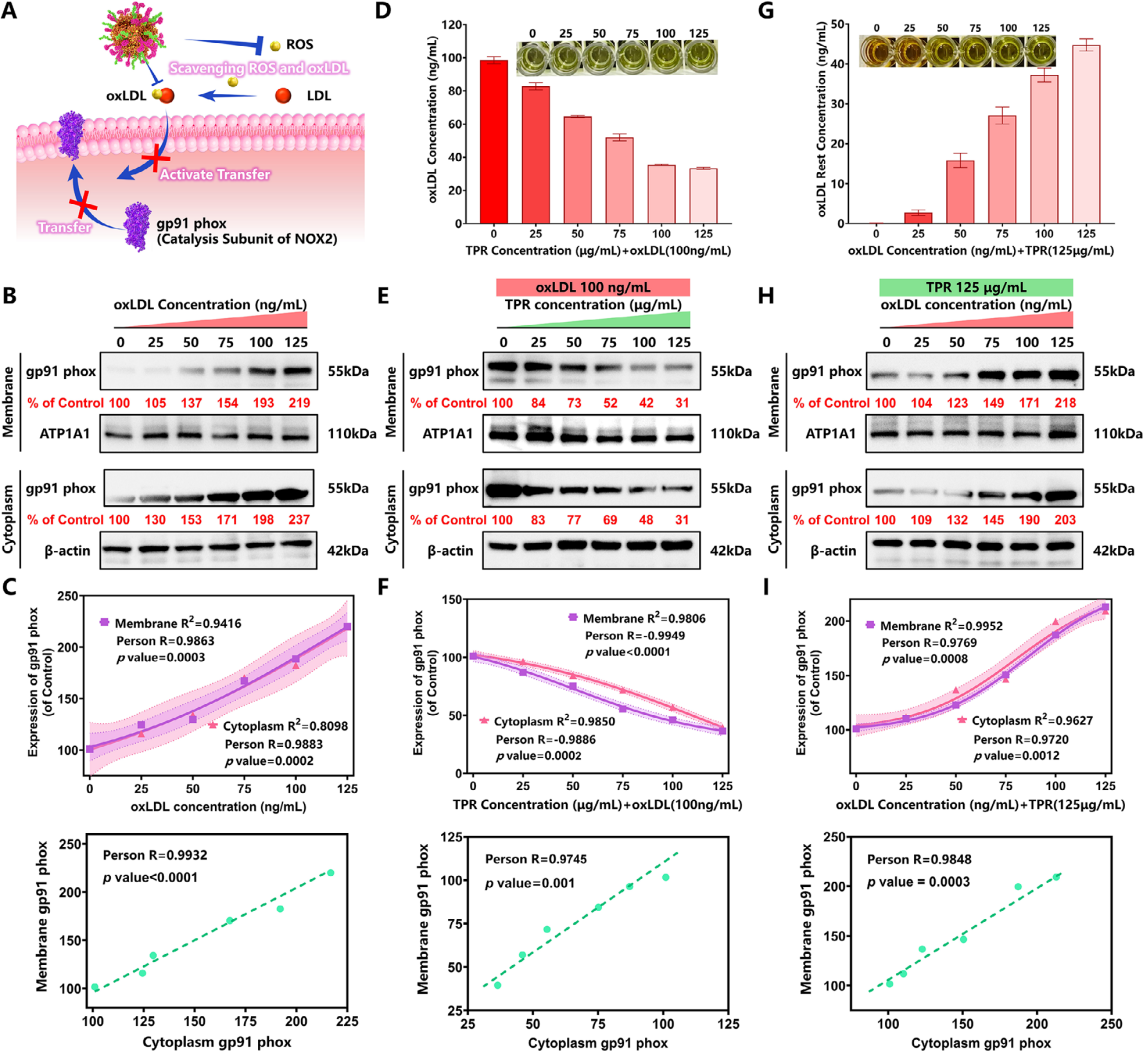
TPR‐LYTACs Inhibit gp91 phox Membrane Translocation by Scavenging Extracellular ox‐LDL. (A) The schematic illustration illustrates the mechanism by which TPR‐LYTACs reduce the extracellular oxLDL level to prevent the translocation of the catalytic subunit of NOX2 (gp91phox) from the cytoplasm to the cell membrane. (B) The level of gp91 phox protein expression in the membrane and cytoplasm of different oxLDL concentrations was assessed by Western blot and analysis. (C) The correlation analysis of gp91 phox expression level and different oxLDL concentrations. The correlation analysis of different gp91 phox expression in the membrane and cytoplasm. (D) The residual oxLDL concentration of TPR‐mediated ox‐LDL clearance experiments. (E) The level of gp91 phox protein expression in the membrane and cytoplasm was assessed by Western blot and analysis. (F) The correlation analysis of gp91 phox expression level and different TPR concentrations. The correlation analysis of different gp91 phox expression in the membrane and cytoplasm. (G) The residual oxLDL concentration of TPR (125 µg/mL) mediated different oxLDL concentration clearance experiments. (H) The level of gp91 phox protein expression in the membrane and cytoplasm was assessed by Western blot and analysis. (I) The correlation analysis of gp91 phox expression level and different residual oxLDL concentrations. The correlation analysis of different gp91 phox expression in the membrane and cytoplasm. Mean±SD, *n* = 3.

Current evidence indicates that ox‐LDL present in the synovial fluid of patients with acute GA acted as a key inducer activating NOX2 in macrophages (Figure 5A). In our study, the TPR component of TPR‐LYTACs utilized its capacity to clear ROS and oxidative substances, thereby effectively diminishing the formation of ox‐LDL derived from ROS‐mediated oxidation in the extracellular environment. The reduction subsequently led to the inhibition of the translocation of the NOX2 catalytic subunit gp91 phox from the cytoplasm to the cell membrane.

Macrophages were cultured in complete medium containing different concentrations of ox‐LDL, and the distribution of gp91 phox was examined by Western Blot (Figure 5B). The results demonstrated a gradual increase in gp91 phox expression in both the cell membrane and cytoplasm with rising ox‐LDL concentrations. The relative expression of gp91 phox in the cytoplasm increases with the increase in ox‐LDL concentration, while maintaining an overall positive correlation (Pearson R = 0.9883, *p *= 0.0002, Figure 5C). In contrast, the relative expression of membrane‐localized gp91 phox exhibited a continuous increasing trend, showing a significant positive correlation (Pearson R = 0.9863, *p *= 0.0003). Furthermore, linear regression analysis revealed a significant positive correlation between the relative expression levels of gp91 phox in the cytoplasm and on the membrane (Pearson R = 0.9932, *p *< 0.0001, Figure 5C). The results indicated that ox‐LDL could simultaneously regulate both the expression and subcellular localization of gp91 phox, promoting the translocation of the cytoplasmic catalytic subunit to the cell membrane.

In the TPR‐mediated ox‐LDL clearance experiments, medium containing 100 ng/mL ox‐LDL was pretreated with gradient concentrations of TPR, and residual ox‐LDL levels were measured (Figure 5D). TPR was found to effectively clear ox‐LDL from the medium, achieving approximately 68% clearance at a concentration of 125 µg/mL. As the TPR concentration increased, ox‐LDL clearance was enhanced, accompanied by a gradual decrease in gp91 phox expression, showing a negative correlation between TPR concentration and relative gp91 phox levels (Figure 5E,F). Similarly, under fixed TPR concentration (125 µg/mL) with progressively increasing ox‐LDL concentrations in clearance assays, the ox‐LDL clearance mediated by TPR remained around 65% (Figure 5G). The residual ox‐LDL levels after clearance were positively correlated with gp91 phox expression (Figure 5H,I). These findings further support that TPR‐LYTACs can inhibit the ox‐LDL‐induced translocation of gp91 phox to the cell membrane by reducing extracellular ox‐LDL levels.

### 2.6 TPR‐LYTACs Mediate Targeted Degradation of Membrane gp91phox via a Dual‐Receptor Recognition Mechanism

**FIGURE 6:**
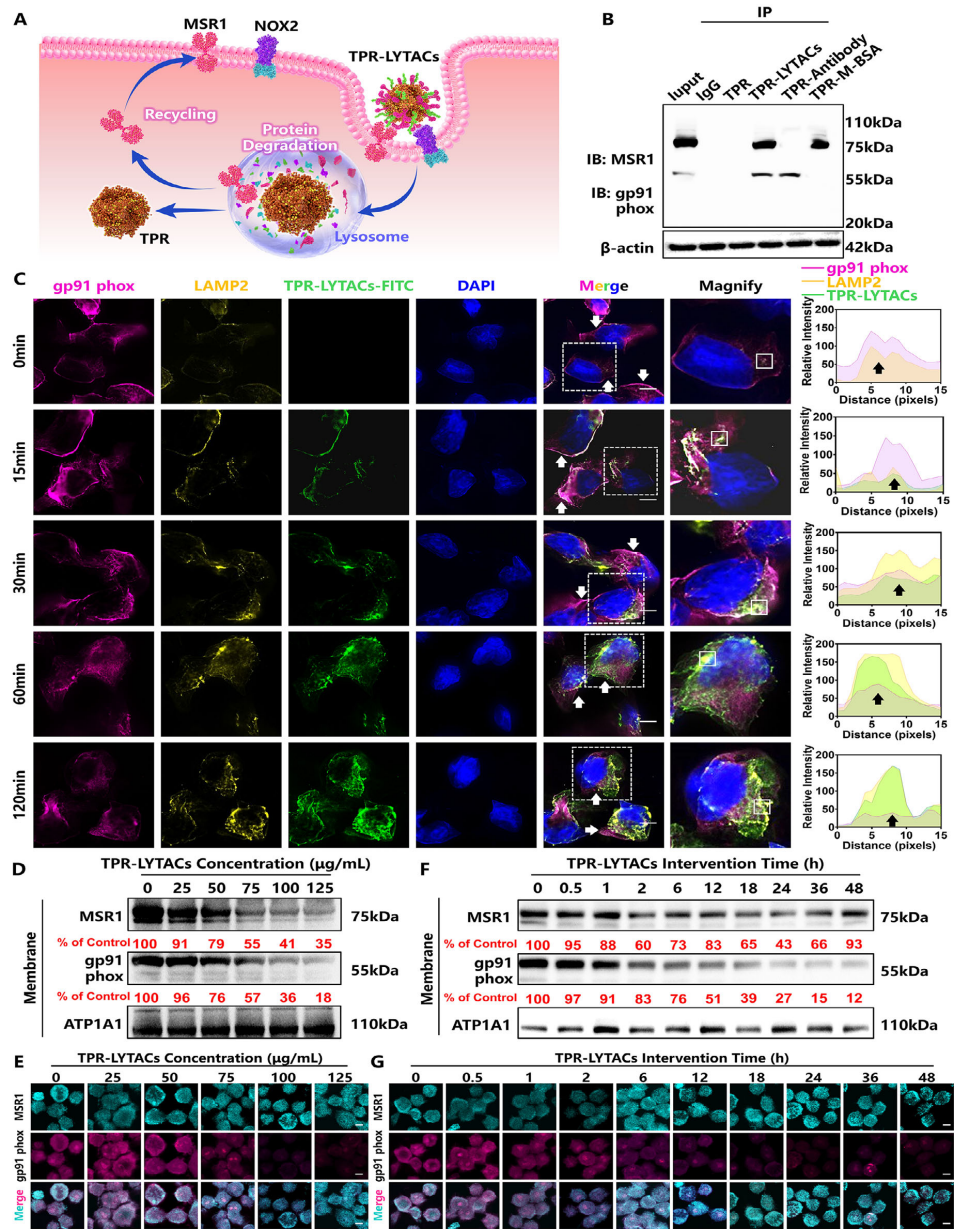
TPR‐LYTACs Mediate Targeted Degradation of Membrane gp91phox via a Dual‐Receptor Recognition Mechanism. (A) The schematic illustration illustrates that the TPR‐LYTACs mediate the clearance of the membrane protein gp91 phox. (B) Co‐immunoprecipitation shows TPR‐LYTACs binding NOX2 and MSR1 protein of cells. Input group is the internal reference control group. IgG group is the negative control group. (C) The immunofluorescence analysis of TPR‐LYTACs with FITC, lysosome, and gp91 phox localization. Scale bar 20 µm. DAPI exposure time 10 ms. gp91 phox exposure time 70 ms. LAMP2 exposure time 30 ms. TPR‐LYTAC‐FITC exposure time 25 ms. (D) The level of MSR1 and gp91 phox protein expression in the membrane of different TPR‐LYTACs concentrations for 24 h was assessed by Western blot and analysis. (E) The immunofluorescence images of MSR1 and gp91 phox protein expression in the membrane and cytoplasm through different TPR‐LYTACs concentrations intervention for 24 h. Scale bar 10 µm. (F) The level of MSR1 and gp91 phox protein expression in the membrane with 125 µg/mL TPR‐LYTACs at different intervention timeline points was assessed by Western blot and analysis. (G) The immunofluorescence images of MSR1 and gp91 phox protein expression in the membrane and cytoplasm with 125 µg/mL TPR‐LYTACs at different intervention timeline points. Scale bar 10 µm. DAPI exposure time 10 ms. gp91 phox exposure time 70 ms. MSR1 exposure time 50 ms.

Based on the dual‐targeting capability of TPR‐LYTACs, the clearance effect on the membrane protein gp91 phox was investigated (Figure 6A). TPR‐LYTACs were directed by MBSA to the endocytic MSR1 receptor and simultaneously bound membrane‐localized gp91 phox via antibody recognition. The dual‐targeting collectively mediated the internalization and subsequent lysosomal degradation of the resultant complex. During the process, the MSR1 receptor was recycled back to the membrane following ligand release, whereas degradation of gp91 phox led to a reduction in membrane NOX2 levels. Concurrently, the degradation‐resistant TPR core was able to dissociate within the lysosome and be released into the cytoplasm, where it continued to exert its antioxidant function, thereby enabling sustained intracellular antioxidative effects.

The targeting binding capability of TPR‐LYTACs‐related materials to MSR1 and gp91 phox proteins produced by M1 macrophages was evaluated using co‐immunoprecipitation. As shown in Figure 6B, the presence of both MSR1 and gp91 phox was detected in the input group containing total protein, which confirmed reliable sample quality. When control IgG was used for precipitation, no significant bands for MSR1 or gp91 phox were observed, thus excluding the possibility of nonspecific binding. In the key experimental groups, no binding signals to either MSR1 or gp91 phox were detected in the precipitate of the TPR core alone, confirming that TPR itself lacks direct interaction with these target proteins. In contrast, distinct bands for both MSR1 and gp91 phox were simultaneously detected in the precipitate of the full TPR‐LYTACs molecule. The findings provided strong evidence that TPR‐LYTACs successfully mediated the formation of a ternary complex with these two key receptors. In the precipitate of TPR‐Antibody, only gp91 phox was detected, while the MSR1 signal was absent. Correspondingly, the precipitate of TPR‐MBSA showed only MSR1 with no gp91 phox signal. The finding provides direct evidence clarifying the molecular mechanism by which TPR‐LYTACs exert their therapeutic function through bridging and co‐internalizing these two receptors.

The TPR‐LYTACs with FITC label (TPR‐LYTACs‐FITC) were used to monitor the intracellular distribution dynamics, and the localization of the lysosomal marker protein LAMP2 and the NOX2 subunit gp91 phox was analyzed by immunofluorescence staining (Figure 6C and Video S1). Before TPR‐LYTACs addition (0 min), abundant gp91 phox localization was observed on the membrane of M1 macrophages with a weak LAMP2 positive signal, suggesting limited lysosomal distribution at this stage. Interestingly, following 15 min of incubation, distinct FITC‐labeled signals began to appear in the membrane region, indicating TPR‐LYTACs enrichment on the cell surface. Concurrently, a gradual increase in LAMP2 fluorescence intensity was observed, implying elevated lysosomal numbers or activity. Colocalization analysis revealed overlapping signals of gp91 phox and TPR‐LYTACs in localized areas, demonstrating that the internalization process had begun. With prolonged incubation and increased intracellular accumulation of TPR‐LYTACs, the gp91 phox signal on the cell membrane progressively diminished (indicated by white arrows). Additionally, the overall immunofluorescence intensity of gp91 phox in the cytoplasm showed a declining trend, suggesting the degradation of gp91 phox upon TPR‐LYTACs treatment.

Simultaneously, the interrelationship between MSR1 and gp91 phox on the macrophage membrane was investigated. The macrophage cells were first treated with gradient concentrations of TPR‐LYTACs for 24 h (Figure 6D,E). Western blot analysis demonstrated a dose‐dependent decrease in the membrane expression levels of both MSR1 and gp91 phox as TPR‐LYTACs concentration increased. The reduction in gp91 phox expression was further verified through immunofluorescence localization assays. Notably, the fluorescence intensity of MSR1 in the cytoplasm was not seemly diminished with increasing TPR‐LYTACs concentration, which diverged from the initial hypothesis. To further investigate our observation, macrophages were continuously treated with 125 µg/ml TPR‐LYTACs, and membrane protein expression was assessed at different timeline points (Figure 6F,G and Figure S4A,S4). Western blot and flow cytometry results indicated that MSR1 expression gradually decreased within the first 6 h, followed by a transient recovery at the 6‐h time point, suggesting a potential compensatory upregulation mechanism. The expression reached its lowest level at 24 h, while partial recovery was observed at 48 h, indicating reversible membrane localization with substantial regulatory potential. In contrast, membrane expression of gp91 phox showed a continuous decline throughout the treatment period without significant re‐expression. Immunofluorescence analysis further revealed the spatiotemporal regulation characteristics of MSR1, as membrane localization weakened during the first 6 h while total fluorescence intensity remained relatively stable during the 6‐h to 48‐h period, suggesting minimal protein degradation. The membrane fluorescence intensity of MSR1 displayed an initial decrease followed by subsequent recovery, indicating internalization and membrane recycling during this phase. Meanwhile, the fluorescence signal of gp91 phox progressively shifted from the membrane to the cytoplasm over time with concurrent reduction in overall intensity, demonstrating not only altered localization but also substantial protein degradation. The two proteins showed differential responses, suggesting distinct regulatory mechanisms. Their coordinated action through the common LYTACs approach further highlighted synergistic and progressive interactions in modulating macrophage immune function.

### 2.7 TPR‐LYTACs Suppress the Intracellular Combination between the Regulatory and Catalytic Subunits

**FIGURE 7:**
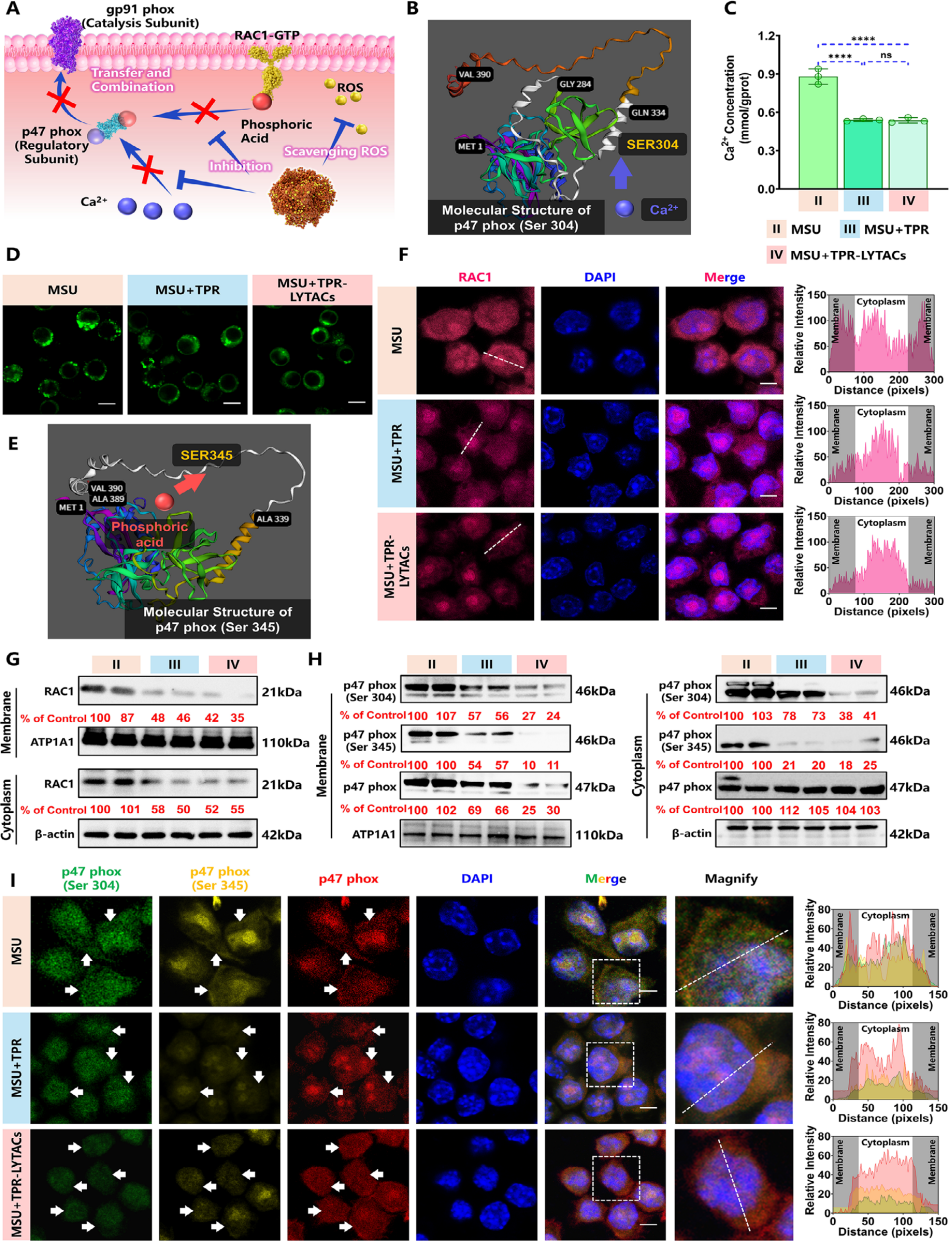
TPR‐LYTACs Mediate Targeted Degradation of Membrane gp91phox via a Dual‐Receptor Recognition Mechanism. (A) The schematic mechanism illustration of inhibiting NOX2 complex activation and assembly through TPR. (B) The diagram of binding site of Ca^2+^ on p47 phox protein (Ser 304). (C) The Ca^2+^ concentrations in the cytoplasm of each group. (D) The Ca^2+^ fluorescence images in each group. Exposure time 30 ms. Scale bar 50 µm. (E) The diagram of binding site of phosphoric acid on p47 phox protein (Ser 345). (F) The immunofluorescence and membrane localization analysis of RAC1. Scale bar 10 µm. RAC1 exposure time 80 ms. (G) The level of RAC1 protein expression in the membrane and cytoplasm with TPR‐LYTACs was assessed by Western blot and analysis. (H) The level of p47 phox, p47 phox (ser 304), and p47 phox (ser345) protein expression in the membrane and cytoplasm with TPR‐LYTACs was assessed by Western blot and analysis. (I) The immunofluorescence and membrane localization analysis of p47 phox, p47 phox (ser 304), and p47 phox (ser345) protein. Scale bar 10 µm. DAPI exposure time 10 ms. P47 phox, ser304, and ser345 exposure time 70 ms. Mean±SD, *n* = 3, ^*^ *p *< 0.05, ^**^ *p *< 0.01, ^***^ *p *< 0.001, ^****^ *p *< 0.0001, ns *p *≥ 0.05.

The catalytic activity of NOX2 required the association of regulatory subunit p47phox and gp91phox for fully functional complex formation. The process depended on the phosphorylation‐induced activation of p47 phox and the unblock of its autoinhibited conformation (Figure 7A).

Among the regulatory mechanisms, Ca^2+^ was shown to induce a conformational change in p47 phox (Ser304), thereby relieving its autoinhibition [41]. At the same time, TPR was found to reduce cytosolic Ca^2+^ levels moderately, promoting the restoration of the autoinhibitory conformation of p47 phox (Figure 7B). These combined effects led to a decrease in the binding capacity between p47 phox and gp91 phox. Measurement of cytosolic Ca^2+^ levels demonstrated that both TPR and TPR‐LYTACs were able to lower Ca^2+^ concentrations compared with the MSU group, and this outcome was further supported by cellular Ca^2+^ fluorescence staining (Figure 7C,D, and Figure S5A).

The membrane anchoring of RAC1‐GTP provides the necessary phosphate source for the phosphorylation of p47 phox (Ser 345), and the anchoring and phosphorylation activation process was inhibited by the function of TPR (Figure 7E) [41]. Immunofluorescence analysis of RAC1 demonstrated that both TPR and TPR‐LYTACs reduced RAC1‐GTP membrane anchoring, while the fluorescence intensity in the cytoplasm correspondingly decreased (Figure 7F and Figure S5B). These findings were consistent with Western blot results, which showed comparable reductions in RAC1 protein expression in both membrane and cytoplasmic fractions in the TPR and TPR‐LYTACs groups compared to the MSU group (Figure 7G).

The expression levels of p47 phox (Ser 304), p47 phox (Ser 345), and total p47 phox in both membrane and cytoplasmic fractions were simultaneously examined by Western blot and immunofluorescence colocalization (Figure 7H,I, and Figure S5C). Compared with the MSU group, the total p47 phox expression on the membrane was reduced in both TPR and TPR‐LYTACs groups, with a more pronounced reduction observed in the TPR‐LYTACs group. A similar trend was found for p47 phox (Ser 304) and p47 phox (Ser 345) expression, indicating that TPR significantly decreased the phosphorylation activation and conformational changes of membrane‐localized p47 phox. In contrast, TPR‐LYTACs further reduced p47 phox expression on the membrane by transporting the gp91phox‐bound p47 phox subunits to lysosomes for degradation. Cytoplasmic p47 phox levels showed no significant reduction across groups, suggesting that TPR does not directly affect its biosynthesis. Both TPR and TPR‐LYTACs reduced p47 phox (Ser 345) expression relative to the MSU group, and no significant difference was detected between them. The result indirectly confirms that the two agents inhibited RAC1 membrane localization to a similar extent, thereby comparably reducing p47 phox (Ser 345) phosphorylation. In contrast, p47 phox (Ser 304) expression exhibited a distinct pattern. Specifically, TPR‐LYTACs induced a more substantial reduction compared to TPR. The difference might be due to additional inflammatory mechanisms being activated alongside NOX2 degradation. The PI3K‐AKT pathway was a potential candidate that influenced p47 phox conformational changes [42, 43]. Based on these findings, the two interventions act synergistically in time and space to attenuate NOX2 activation and catalysis, providing new mechanistic evidence for TPR‐LYTACs‐mediated regulation of macrophage oxidative stress.

### 2.8 Targeting and Superior Therapeutic Efficacy of TPR‐LYTACs in Acute Gouty Arthritis

**FIGURE 8:**
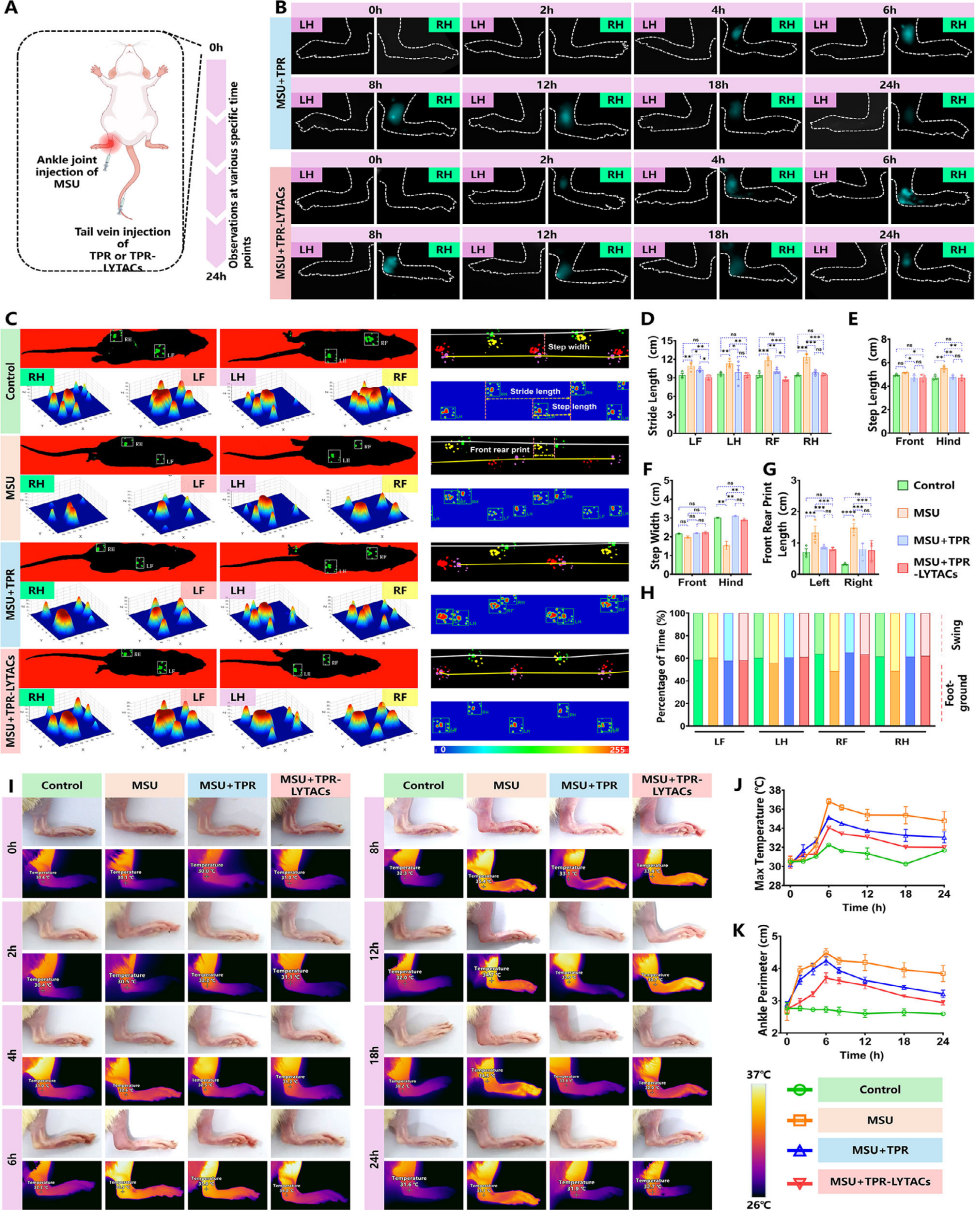
Targeting and Superior Therapeutic Efficacy of TPR‐LYTACs in Acute Gouty Arthritis. (A) Schematic of the experimental design and treatment protocol for the acute GA rat model. (B) In vivo NIR‐II fluorescence imaging analysis in the LH and RH of acute GA rat model with TPR‐LYTACs and TPR. LH, left hind. RH, right hind. (C) The paw contact area and footprint image of gait analysis in each group. LF, left fore. RF, right fore. LH, left hind. RH, right hind. (D) The stride length analysis in each group. (E) The step length analysis in each group. (F) The step width analysis in each group. (G) The front rear print length analysis in each group. (H) The time of front‐ground and swing analysis in each group. (I) The thermal imaging temperature and bright‐field degree of swelling images of RH ankle joints in each group at different timeline points. (J) The temperature analysis of ankle joint thermal imaging in each group. (K) The perimeter analysis of ankle joint swelling in each group. Mean±SD, *n* = 3, ^*^ *p *< 0.05, ^**^ *p *< 0.01, ^***^ *p *< 0.001, ^****^ *p *< 0.0001, ns *p *≥ 0.05.

Based on the above cellular experiment, the in vivo therapeutic efficacy of TPR and TPR‐LYTACs was further evaluated in the acute GA model rats according to the treatment protocol illustrated in Figure 8A. The enrichment kinetics at lesion sites were monitored via NIR‐II FL imaging after intravenous tail injection (Figure 8B). The NIR‐II FL image revealed that TPR accumulation commenced in the gout ankle joint at 4 h postinjection and reached its peak at 8 h. At the same time, no significant enrichment signal was detected in the typical ankle joint used as a control. In comparison, TPR‐LYTACs were detectable in the gout ankle region by 2 h and peaked at 6 h. The pharmacokinetic profile demonstrated faster and stronger targeted enrichment relative to TPR, indicating enhanced targeting capability and retention efficiency at the lesion sites. 6 h after intravenous administration of TPR and TPR‐LYTACs via the tail vein, the ankle joints and major organs (including the heart, liver, spleen, lungs, and kidneys) were collected for NIR‐II fluorescence imaging analysis. Both TPR and TPR‐LYTACs exhibited distinct NIR‐II fluorescence signals in the liver and kidneys, indicating that their metabolism was mainly mediated by the hepatic and renal pathways. Notably, TPR‐LYTACs showed a much stronger NIR‐II fluorescence signal in the GA ankle joint, whereas TPR exhibited only a weak signal at the joint at the same time point (Figure S6A). The joint‐to‐liver NIR‐II fluorescence ratio further demonstrated that TPR‐LYTACs possessed superior targeted accumulation capability in inflamed joints (Figure S6B).

Following the assessment of enrichment of joint sites, functional recovery was evaluated through gait analysis (Figure 8C). The paw contact area of the right hind (RH) was significantly reduced in the MSU group compared to the Control group, suggesting pain‐induced weight‐bearing avoidance. After treatment with TPR and TPR‐LYTACs, the paw contact area was partially restored, with more pronounced improvement observed in the TPR‐LYTACs group. Compensatory changes in motor coordination were also observed in the non‐injected MSU limbs. To further characterize these functional deficits, quantitative gait analysis was performed. The results demonstrated that the MSU group exhibited marked abnormalities in gait relative to the Control group. Specifically, stride length and step length on the affected side were significantly prolonged, reflecting decreased movement willingness and compromised propulsion due to joint pain and swelling (Figure 8D,E). Concurrently, the substantially reduced step width and increased front‐rear print length (Figure 8F,G). The findings indicated a stabilization strategy of compensatory activity of limbs in rats via base of support enlargement, complemented by prolonged swing phase and shortened foot‐ground phase to maximize nonweight‐bearing time (Figure 8H). These coordinated adaptations collectively represent the typical protective limping pattern in acute GA model rats. Although TPR intervention moderately improved all parameters, the TPR‐LYTACs group showed significant restoration of gait dysfunction, bringing stride length, step length, step width, front‐rear print length, and temporal metrics back to nearly normal levels.

To evaluate the treatment effect, the dynamics of ankle joint temperature and swelling were recorded using infrared thermography and bright‐field imaging (Figure 8I). In the control group with PBS injection, no significant fluctuations in RH ankle temperature or swelling were observed over 24 h, confirming that the injection procedure itself did not induce substantial inflammation. In contrast, the RH ankle of rats in the MSU group developed rapid and severe local responses following modeling. The ankle temperature began rising within 1 h and reached its peak at 6 h, coinciding with the time of maximal swelling level (Figure 8J,K). The simultaneous elevation in joint surface temperature and swelling indicated increased local blood flow and a robust acute inflammatory immune response. Both TPR and TPR‐LYTACs treatments effectively delayed the elevation of temperature. The reduced peak values suggested that inflammation‐related signaling pathways or macrophage infiltration may have been suppressed, leading to reduced release of inflammatory mediators and local vasodilation. This control of early inflammatory amplification was further supported by a steady decline in temperature and concurrent reduction in swelling after 6 h in the TPR‐LYTACs treatment. These findings provide comprehensive evidence for the anti‐inflammatory efficacy of TPR‐LYTACs in the acute GA model rats.

### 2.9 Therapeutic Effects of TPR‐LYTACs via Degradation of NOX2 Components and Inhibition of Inflammatory Cascades

**FIGURE 9:**
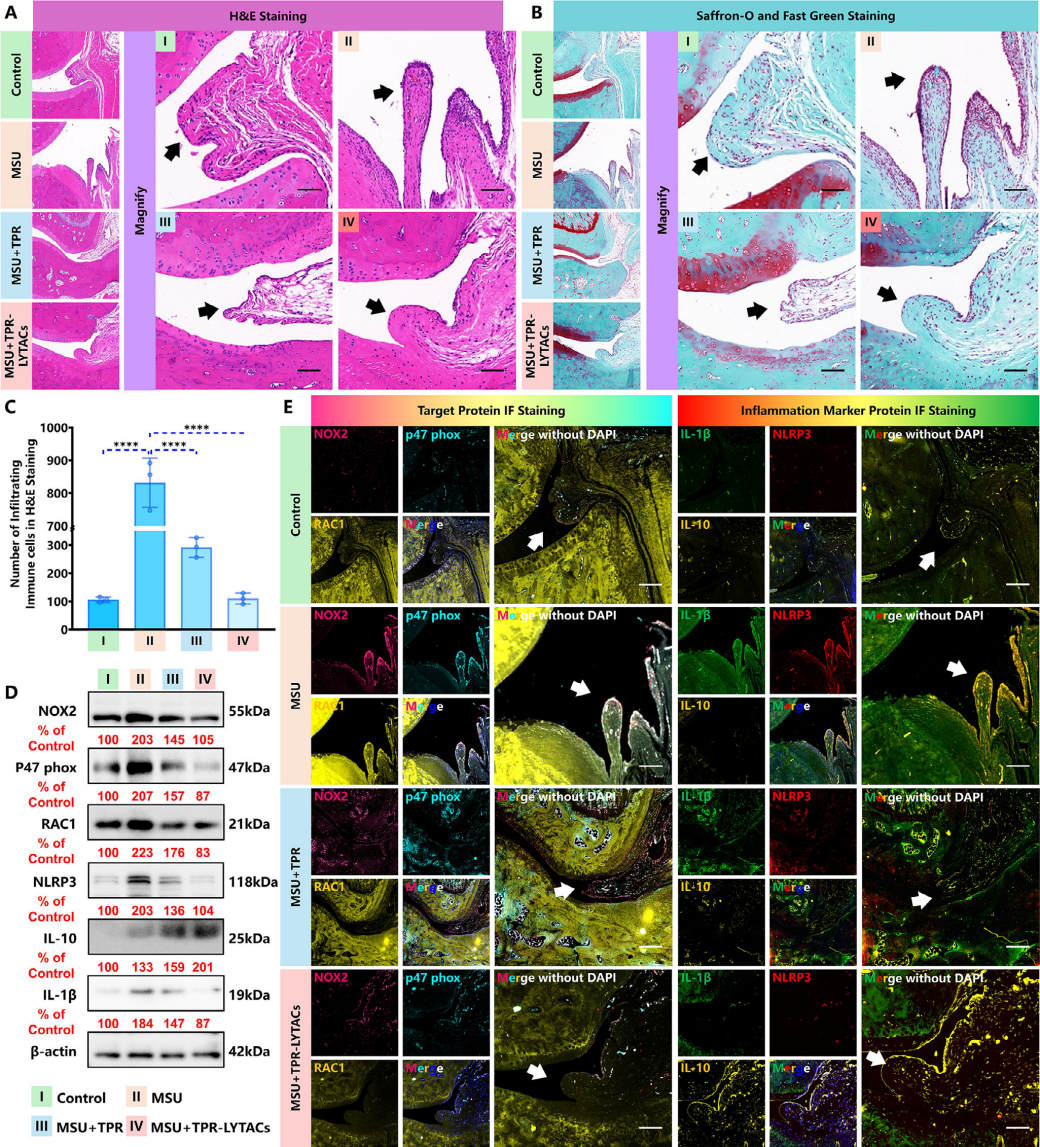
Therapeutic Effects of TPR‐LYTACs via Degradation of NOX2 Components and Inhibition of Inflammatory Cascades. (A) Histopathological assessment of ankle joints by H&E staining. Scale bar 500 µm. (B) Histopathological assessment of ankle joints by safranin O‐fast green staining. Scale bar 500 µm. (C) The analysis of infiltrating immune cells in H&E staining in each group. (D) The level of NOX2, p47 phox, RAC1, and inflammatory markers protein expression in the ankle tissue in each group was assessed by Western blot and analysis. (E) The immunofluorescence image of NOX2, p47 phox, RAC1, and inflammatory markers protein expression in the ankle tissue in each group. Scale bar 500 µm. DAPI exposure time 20 ms. NOX2 exposure time 110 ms. p47 phox exposure time 130 ms. RAC1 exposure time 130 ms. NLRP3 exposure time 70 ms. IL‐10 exposure time 80 ms. IL‐1β Exposure time 80 ms. Mean±SD, *n* = 3, ^****^ *p *< 0.0001.

Inspired by the exciting in vitro results above, we further assessed the pathological alterations and drug intervention effects in the acute GA model by using histological examination. The intact joint structure and a transparent synovial layer were displayed in the normal ankle, as shown by the hematoxylin‐eosin (HE) staining (Figure 9A). Compared with the control group, massive inflammatory cell infiltration in the synovial tissue, hyperplasia, and local tissue edema in the ankle of the MSU group represented typical acute inflammatory pathological changes. After being treated with TPR and TPR‐LYTACs, inflammatory cell infiltration was significantly reduced, and the synovial structure became more regular, with the TPR‐LYTACs group displaying more substantial improvement (Figure 9B). The safranin O‐fast green staining demonstrated largely intact articular cartilage structure across all groups, with smooth surfaces, distinct tidemark organization, and no evidence of proteoglycan loss (Figure 9C). These histological observations indicated that the MSU crystals did not initiate substantial cartilage matrix degradation or structural damage, suggesting the inflammatory process may not have activated critical matrix‐degrading pathways during the short experimental period.

Building upon these pathological observations, the molecular mechanisms of TPR and TPR‐LYTACs in acute GA were analyzed by using Western blot and immunofluorescence. The expression and distribution of key NOX2 complex components and related inflammatory markers were evaluated in joint tissues. Western blot results showed significant upregulation of NOX2 (gp91phox), p47phox, and RAC1 in the MSU group compared to the Control group (Figure 9D). Immunofluorescence localization revealed their predominant aggregation in inflammatory cell infiltration areas, indicating a high degree of colocalization between NOX2 complex activation and inflammatory sites (Figure 9E and Figure S7). The overactivation of the NOX2 complex was closely associated with NLRP3 inflammasome activation and subsequent maturation and release of IL‐1β, concurrent with suppression in the production of anti‐inflammatory cytokine IL‐10. The shift established a pronounced pro‐inflammatory state, ultimately manifesting as joint redness, swelling, heat, pain, and functional impairment. Both TPR and TPR‐LYTACs treatments reversed these changes by downregulating pro‐inflammatory. Furthermore, TPR‐LYTACs exerted synergistic effects by concurrently inhibiting oxidative stress and inflammatory cascades, achieved through efficient degradation of membrane‐associated inflammatory mediators and modulation of signaling pathways. These findings provide a mechanistic basis for the superior therapeutic efficacy of TPR‐LYTACs observed across multidimensional evaluations.

### 2.10 TPR‐LYTACs Biosafety Assessment

**FIGURE 10:**
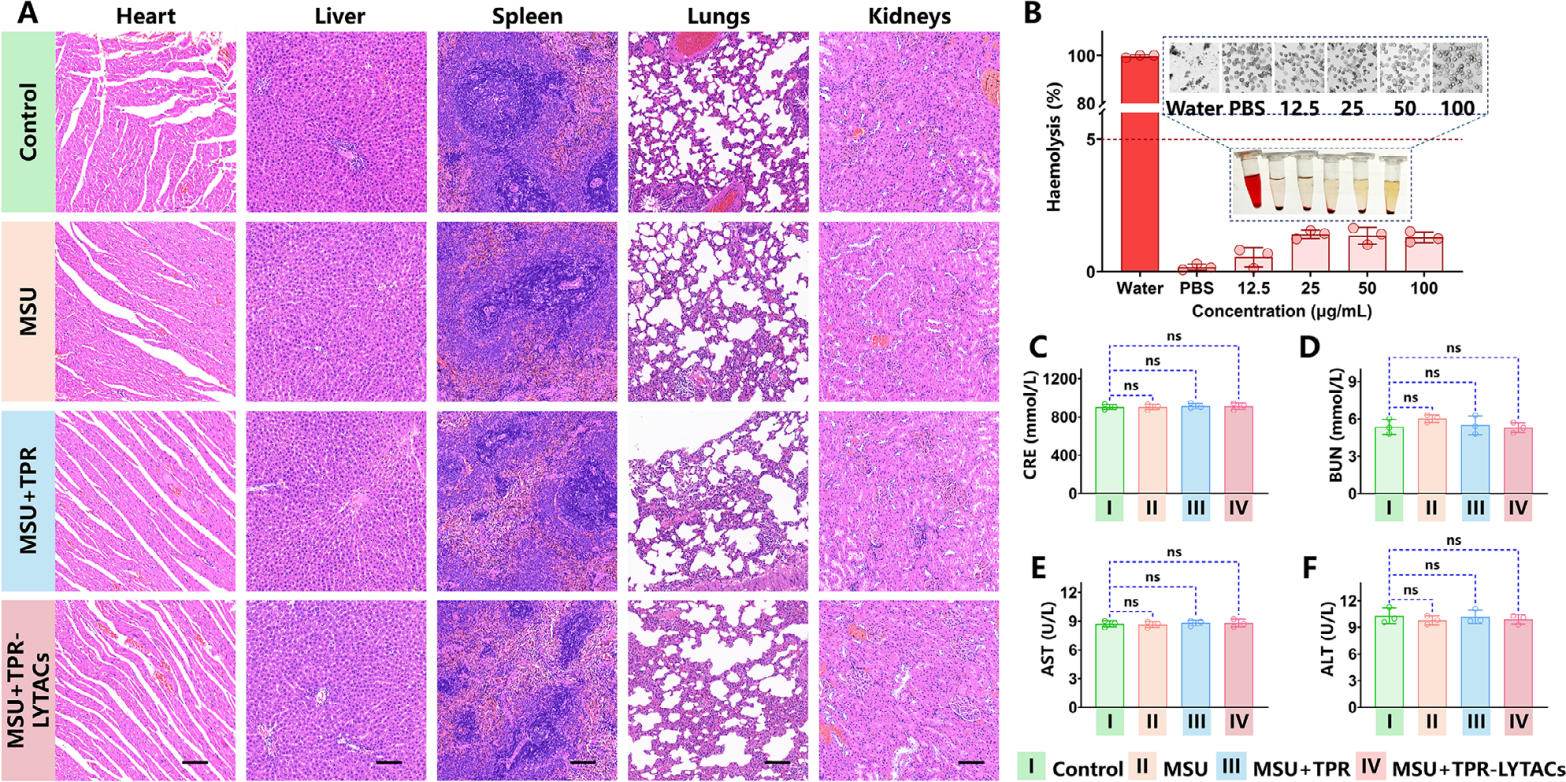
TPR‐LYTACs Biosafety Assessment. (A) Histopathological assessment of major organs by H&E staining. Scale bar 500 µm. (B) Hemolysis assay of TPR gradient on centration. (C) Serum biochemical analysis, including CRE, BUN, AST, and ALT. Mean±SD, *n* = 3, ns *p *≥ 0.05.

To further evaluate the in vivo safety of TPR‐LYTACs, their biocompatibility was systematically assessed through H&E staining of major organs, hemolysis assays, and serum biochemical analysis. The results showed that no obvious histological abnormalities, inflammatory infiltration, or pathological damage were observed in the major organs, including the heart, liver, spleen, lung, and kidney, in any treatment group, and no significant differences were found compared with the control group (Figure 10A). Meanwhile, the hemolysis assay demonstrated that TPR‐LYTACs did not induce noticeable erythrocyte rupture, indicating good hemocompatibility. Based on these results, a concentration of 1 mg/mL TPR‐LYTACs was selected for subsequent tail vein administration in rats (Figure 10B). Further serum biochemical analysis revealed that all measured parameters remained within the normal physiological range, with no obvious differences among the groups. Collectively, these findings indicate that TPR‐LYTACs exhibit favorable in vivo safety and do not cause significant toxic effects on major organ functions or the blood system (Figure 10C–E). Combined with their good tissue tolerance, hemocompatibility, and physiological stability, TPR‐LYTACs are suggested to possess strong potential for in vivo application, providing important support for their further investigation and translational development in disease treatment.

## 3 Conclusions

In summary, the TPR‐LYTACs were demonstrated to exhibit significant comprehensive therapeutic advantages in an acute GA. The effective inhibition of NOX2 complex assembly and activation was achieved by TPR‐LYTACs, thereby blocking the explosion generation of ROS at its source. At the same time, pre‐existing ROS were directly scavenged by the nano‐core, consequently breaking the vicious cycle between oxidative stress and inflammatory response. In acute GA rats, the TPR‐LYTACs resulted in marked improvements in joint swelling, gait function, and inflammatory cell infiltration by effectively regulating the NOX2/NLRP3/IL‐1β signaling axis. This study provides a brand new perspective for overcoming the limitations of conventional antioxidants and single‐target strategies. Taken together, the TPR‐LYTACs achieve a dual‐track intervention that integrates the source of ROS inhibition with the microenvironment clearance, while also serving as a representative phyto‐nanotheranostics platform for the precise treatment of gout and other inflammatory diseases.

## 4 Experimental Section

Materials and experimental details are provided in the Supporting Information. All animal studies were performed in the Animal Experiment Center of Shanxi Medical University, and the procedures involving experimental animals were in accordance with protocols approved by the Institutional Animal Care and Use Committee of the Animal Experiment Center of Shanxi People's Hospital (No. 2025–182, Taiyuan, China).

### 4.1 Statistical Analysis

Statistical analyses were performed using SPSS software (version 25, IBM, Armonk, NY, USA). Data were expressed as mean ± standard deviation (SD). Statistical significance was assessed via the two‐tailed unpaired Student's *t*‐test and One‐way ANOVA. All *n*‐values per group were reported in the figure legends. No statistical methods were used to predetermine sample size in the study. The *p *< 0.05 was considered to be statistically significant (^*^ *p *< 0.05, ^**^ *p *< 0.01, ^***^ *p *< 0.001, ^****^ *p *< 0.0001), and a *p *≥ 0.05 indicated that the differences were not significant (ns).

## Author Contributions

**Mengting Gao**: validation, formal analysis. **Yingying Wang**: conceptualization, investigation, writing – original draft, methodology, validation, visualization, software, formal analysis. **Jian Zhang**: conceptualization, investigation, methodology, validation, writing – original draft, funding acquisition, visualization, formal analysis. **Lujie Yu**: conceptualization, investigation, validation, formal analysis, data curation. **Chunmei Jiang**: conceptualization, investigation, validation, formal analysis, supervision. **Qin Liu**: conceptualization, validation, formal analysis, supervision, data curation. **Shutong Wu**: methodology, validation, software, supervision. **Haoyu Liu**: conceptualization, investigation, validation, formal analysis, software. **Ruiping Zhang**: conceptualization, investigation, funding acquisition, writing – original draft, writing – review and editing, supervision, data curation. **Yaohua Chen**: conceptualization, investigation, validation, formal analysis, supervision. **Huifang Hao**: investigation, validation, formal analysis, supervision. **Rong Dai**: conceptualization, methodology, supervision, data curation, project administration. **Lingling Wei**: investigation, funding acquisition, validation, visualization, software, formal analysis, supervision. **Xiaochun Zheng**: visualization, validation, software, formal analysis. **Ziliang Zheng**: conceptualization, investigation, writing – review and editing, project administration, resources, supervision, data curation, funding acquisition. **Weiwei Kang**: validation, formal analysis, supervision.

## Conflicts of Interest

The authors declare no conflicts of interest.

## Supporting information




**Supporting File 1**: advs75889‐sup‐0001‐SuppMat.docx.

**Supporting File 2**: advs75889‐sup‐0002‐VideoS1.mp4.

## Data Availability

The data that support the findings of this study are available from the corresponding author upon reasonable request.
