# Supplementary material for: NIR‐II‐Trackable LYTACs Phyto‐Nanotheranostics for Source‐Microenvironment Dual‐Track ROS Regulation in Acute Gouty Arthritis
Source: Adv Sci (Weinh). 2026 May 30;13(48):e75889. doi: 10.1002/advs.75889 (PMC13335956; doi:10.1002/advs.75889)
Supplement: Supplementary file 1 [file ADVS-13-e75889-s001.docx]

**Supporting Information**

**Materials and Methods**

**Experimental Section**

**Materials**

All commercial reagents and solvents were used as received without further purification unless otherwise noted. Tea polyphenol (#D051741-5g), Rutin (#JX575587), Maleic anhydride (#EB000069), and N-Hexadecyltrimethylammonium chloride (#E010074) were purchased from Energy Chemical, (Shanghai, China). N,N,N′,N′-Tetramethylethylenediamine (TEMED) (#T105497) was purchased from Shanghai Aladdin Biochemical Technology Co., Ltd, (Shanghai, China). Fetal Bovine Serum (FSP500) was purchased from ExCell Bio, (Shanghai, China). Cell Counting Kit-8 (CCK-8, #C6005) was purchased from Suzhou NCM Biotechnology Co., Ltd, (Suzhou, China). Coenzym II NADP(H) Content Assay Kit (#BC1105), Sodium Dodecylsulfate (SDS, #S8010), Tris (Hydroxymethyl) Aminomethane (#T8061), Glycine (#G8200), oxLDL (#IO1300), Bovine Serum Albumin (BSA, #A8010), FITC (#IF0081), and CY5 (#S1053) were purchased from Beijing Solarbio Science & Technology Co., Ltd (Beijing, China). DMEM (#G4515-500ML), PBS (G4202), Reactive Oxygen Species Assay Kit (DCFH-DA, #G1706-100T)Dihydroethidium (DHE, #G1746-100T), Tyramide signal amplification (TSA) PLus Fluorescence Five Label Six Color Staining Kit (#G1256-50T), 30% Acr-Bis (29:1) (#G2004-500ML), 1.5 M Tris-HCl (pH 8.8) (#G2053-100ML), 1 M Tris-HCl (pH 6.8) (#G2054-100ML), Marker IV (8-200 kDa) (#G2083-250UL), HRP conjugated Goat Anti-Rabbit IgG (H+L) (#GB23303), HRP conjugated Goat Anti- Mouse IgG (H+L) (#GB23301), Recombinant Anti-beta Actin antibody (Rabbit mAb) (#GB15003-100), Hoechst 33342 (G1127-1ML), and Lyso-Tracker Red (#G1741-50UL) were Wuhan Servicebio Technology (Wuhan, China). RIPA complete lysis buffer (#P0038-100ml), QuickBlock^TM^ universal protein-free blocking, Fluo-4 AM (calcium ion fluorescent probe, 2mM, #S1056), and antibody dilution buffer (#P0270-500ml) were purchased from Beyotime of Biotechnology (Shanghai, China). Cocktail (#SJ-MK0001-B) was purchased from SparkJade Biotechnology Co., Ltd, (Shandong, China). PO-1 (#P991796) was purchased from Macklin Inc, (Shanghai, China). ATP1A1 (#83191-6-RR), NOX2 (gp91 phox) Polyclonal antibody (#19013-1-AP), RAC1 Monoclonal antibody (66122-1-Ig), Rabbit IgG control Polyclonal antibody (#30000-0-AP) were purchased from Proteintech Group, Inc. (Wuhan, China). p47-phox Rabbit pAb (#YT3520), p47-phox (Phospho Ser304) Rabbit pAb (#YP0204), and p47-phox (Phospho Ser345) Rabbit pAb (#YP0828) were purchased from Immunoway Biotechnology Co., Ltd, (Suzhou, China). Mouse oxLDL (Oxidized Low Density Lipoprotein) ELISA Kit (#ELK7649) was purchased from ELK Biotechnology Co., Ltd, (Wuhan, China).

**Instruments**

DLS measurements and zeta potential analysis were performed on a Zetasizer Nano-ZS 2000 (Malvern Instruments, USA) at 25 °C. TEM measurements were performed on the HT7800 electron microscope at an acceleration voltage of 120 kV (Hitachi, Japan). UV-vis spectrum was performed on TU-1901 dual-beam UV-vis spectrophotometer (PerkinElmer, USA). FTIR spectroscopy was carried out using the KBr pellet technique on a spectrophotometer (Bruker Tensor II, Germany) to identify and quantify the relative abundance of bonds. Immunofluorescence images and live cell video were captured using the MICA fully integrated live cell imaging and analysis platform (Leica, Germany). Western blot was performed on the SPW-DM vertical electrophoresis system and SCG-W5000 PLUS imaging system (Servicebio, China). Flowcytometric assays were performed on a FongCyte^TM^ S flow cytometry (Challenbio, China). The RNA transcriptome sequencing was conducted by Novogene, using the Illumina RNA platform.

**Synthesis of TPR-LYTACs**

The preparation of the target product TPR-LYTACs was initiated with the synthesis of the precursor A-Rutin. Rutin was completely dissolved in anhydrous ethanol. The solution was placed in a 60 ℃ water bath under continuous stirring with HCl solution for 4 h. The resulting product was obtained as A-Rutin powder by freeze-drying. Subsequently, TPR nanoparticles were assembled. TP was dissolved in deionized water, followed by the slow addition of a pre-prepared A-Rutin solution. After thorough mixing, cetyltrimethylammonium chloride solution, acting as a surfactant, and ammonia water, serving as a condensation catalyst, were sequentially introduced. The entire assembly process was completed under room temperature stirring for a total of approximately 4.5 h. The crude product was purified and concentrated three times using a 100 kDa molecular weight cutoff ultrafiltration centrifuge tube at 3000 rpm, washed with ultrapure water, and then freeze-dried to obtain TPR nanoparticles. Next, protein complexation and functionalization were performed. First, TPR and bovine serum albumin (BSA) were combined at a 10:1 mass ratio in deionized water with overnight stirring at room temperature to form a stable TPR-BSA complex. Unreacted BSA was removed using a 30 kDa ultrafiltration centrifuge tube at 3000 rpm, after which the complex was redispersed in sodium pyrophosphate buffer (pH 9.0). Under an ice-water bath, 1 M maleic anhydride, dissolved in dioxane, was slowly added dropwise. The reaction system pH was consistently maintained at 9.0 by continuous addition of sodium hydroxide solution, followed by incubation on ice for 5 min to achieve maleylation of BSA. The resulting TPR-MBSA was purified by extensive dialysis against phosphate buffer (pH 7.4) at 4 °C. Finally, the targeting ligand was conjugated. The TPR-M-BSA were dispersed in PBS, and a mixed solution of EDC and NHS was added to activate the carboxyl groups for 1 h on ice. The activated product was purified and collected using a 30 kDa ultrafiltration centrifuge tube at 3000 rpm, washed with PBS, and then redispersed in 350 mL PBS (pH 5.8). It was incubated with NOX2 (gp91 phox) antibody overnight at room temperature to achieve covalent coupling. The final product, TPR-LYTACs, was purified using the same centrifugation and washing procedure and was stored at 4 °C for subsequent use.

**Cell Culture**

RAW264.7 cells (#STCC20020P) and HUVECs (#STCC12103P) were obtained from the Wuhan Servicebio Technology (Wuhan, China). The cells were cultured in high-glucose DMEM medium supplemented with 10% fetal bovine serum (FBS) and maintained at 37 °C in a humidified atmosphere containing 5% CO_2_.

**Cellular Uptake Assessment**

RAW264.7 and HUVECs cells were seeded in 96-well plates at a density of 1.0 × 10⁵ cells per well and pretreated overnight with MSU (100 μg/mL). After washing with PBS, the cells were treated with TPR, TPR-MBSA, or TPR-LYTACs at different intervention time points. Following incubation and careful washing with PBS buffer, cellular uptake was visualized using an NIR-II fluorescence microscope.

**Cytotoxicity Assessment**

The cytotoxicity of TPR-LYTACs was assessed using a standard CCK-8. RAW264.7 and HUVECs cells were seeded at 1.0*10^5^ cells per well and treated with different concentrations of TPR or TPR-LYTACs. Subsequently, 10 μL of CCK-8 solution was added to each well and incubated at 37 °C for 30 min. The absorbance was then measured at 450 nm to assess cell viability.

**Intracellular ROS Evaluation**

Total ROS and its subtypes (H_2_O_2_ and •O_2_⁻) were detected using fluorescent probes DCFH-DA, DHE, and PO-1. RAW264.7 cells were seeded in 96-well plates and primed with MSU (100 μg/mL) for 24 h, and then the cells were treated with TPR or TPR-LYTACs for 24 h. The cells were then incubated with fluorescent probes DCFH-DA, DHE, or PO-1 (each at 5 μM) for 20 min. Fluorescence signals were measured by fluorescence microscope and flow cytometry at their respective optimal excitation wavelengths: 488 nm for DCFH-DA, 594 nm for DHE, and 535 nm for PO-1.

**Immunofluorescence**

Multi-marker fluorescence staining of cells and tissue sections required the TSA PLus Fluorescence Five Label Six Color Staining Kit. RAW264.7 cells were seeded on coverslips in confocal dishes and primed with MSU (100 μg/mL) and TPR relative nanomaterials for 24 h. After treatment, the cells were fixed with 4% paraformaldehyde and permeabilized for immunofluorescence staining. Following blocking, the cells were incubated overnight at 4°C with the following primary antibodies: rabbit anti-mouse antibodies against NOX2 (gp91 phox), p47 phox, p47 phox (Ser304 and Ser345), LAMP2, NLRP3, IL-10, and IL-1β; along with mouse-derived antibodies against RAC1 and MSR1. Cell nucleus were counterstained with DAPI or Hoechst 33342. Fluorescence images were acquired using the confocal microscope and processed with the accompanying software for contrast enhancement.

**Western blot Analysis**

The cells and rat ankle tissue were collected and lysed to extract total proteins. Protein samples were denatured, separated by SDS-PAGE, and transferred onto a PVDF membrane. The membrane was incubated at room temperature for 8 hours with primary antibodies against NOX2, MSR1, phox, gp91, gp91 phox, RAC1, p47 phox, p47 phox (Ser304 and Ser345), NLRP3, IL-10, and IL-1β, followed by incubation with a corresponding secondary antibody for 1 h at room temperature. Protein bands were visualized using a chemical luminescence imaging instrument.

**Immunoprecipitation**

Cells were lysed using IP lysis buffer supplemented with a protease inhibitor cocktail. The cell lysates were then incubated with the TPR relative nanomaterials at room temperature for 2 h to form immunocomplexes. After incubation, the TPR relative nanomaterials were washed thoroughly with IP wash buffer. The bound complexes were eluted using SDS sample loading buffer, and 5× protein loading buffer was added. The samples were heated at 95 °C for 5 min, and the supernatant was collected for subsequent Western blot analysis.

**Assessment of Intracellular Levels of NADP^+^ and NADPH**

The cellular redox state was evaluated by measuring the intracellular levels of NADP^+^, NADPH, and the NADP^+^/NADPH ratio. After treatments, cells were collected and rapidly lysed. The cell lysates were immediately divided into two aliquots: one for total NADP (NADP+, NADPH) measurement and the other for NADPH-only measurement after selective decomposition of NADP+. Both aliquots were then centrifuged, and the supernatants were collected. Subsequently, each supernatant was incubated with a specific enzyme cycling reaction mix at 37 °C for a defined period, protected from light. The reaction was stopped, and the absorbance was measured at 340 nm using a microplate reader. The concentrations of NADP^+^ and NADPH were calculated based on respective standard curves. The NADP^+^/NADPH ratio was then determined from these calculated values.

**Evaluation of Pro-inflammatory and Pro-oxidant Load**

The quantified or varying concentrations of oxLDL and TPR were added to the culture medium, and the remaining concentration of oxLDL was measured after clearance. The resulting supernatant, along with standards and reagents, was sequentially added to the microplate according to the manufacturer's instructions. The reaction mixture was incubated at 37°C for a specified duration. The absorbance (OD value) was then measured at 450 nm using a microplate reader. The oxLDL concentration was calculated based on a standard curve. Concurrently, the rest concentration, indicative of the remaining substrate after the cellular oxidative reaction, was determined to evaluate the pro-oxidant load comprehensively.

**Calcium Homeostasis Assessment**

Cells were collected, and the pellet was washed twice with PBS via centrifugation (2,000 rpm, 5 min). After discarding the supernatant, the pellet was resuspended in 0.5 mL of deionized water and subjected to sonication for disruption. The lysate was then centrifuged (2,000 rpm, 10 min), and the resulting supernatant was transferred to a healthy plate and mixed with the working solution. Following 5 min incubation, the optical density was measured at 610 nm to determine the calcium content. The Ca^2+^ fluorescent images of cells were detected by using the fluo-4 AM calcium ion fluorescent probe.

**Establishment of the Acute Gouty Arthritis Rat Model**

Healthy adult male Sprague-Dawley rats (weight 180-220 g) were used in this experiment. All rats were housed under standard laboratory conditions (room temperature 22 ± 2 °C, 12‑h light/dark cycle) with free access to standard feed and water. Monosodium urate crystals were suspended in sterile phosphate-buffered saline to prepare a suspension at the required concentration (typically 25-50 mg/mL). On the day of modeling, the cavity of the right ankle joint was located, and 0.1 mL of the MSU suspension (containing 25 mg/mL MSU) was slowly injected into the joint space. After injection, the joint was gently moved to ensure even distribution of the suspension. Rats in the blank control group were injected with an equal volume (0.1 mL) of sterile PBS into the right ankle joint cavity under identical conditions.

**In Vivo NIR-II FL Imaging**

To detect the biodistribution of TPR and TPR-LYTACs in vivo, the CIA mice were intravenously injected with TPR and TPR-LYTACs. After tail vein injection, the images of NIR-II FL imaging were obtained at different points by II 900/1700 in vivo imaging system (Suzhou NIR Optics Co., Ltd., China).

**In Vivo Therapeutic Efficacy Evaluation**

The rats were randomly divided into four groups (n = 6) with distinct treatments as follows: PBS, MSU, MSU+TPR, and MSU+TPR-LYTACs to evaluate the therapeutic effects on the ankle joint. 0.1 mL of different solutions was intravenously injected into each rat. During the treatment, the photographs of paws and infrared thermography of each rat were recorded to evaluate the disease progression of acute GA.

**Gait Analysis**

The gait analysis for the rats with different treatments was performed by the VisuGait Small Animal Gait analysis system (XR-FP101, Shxinruan Information Technology Co., Ltd., China), and the gait parameters of freely moving mice were recorded. Briefly, the rats were placed at one end of the walkway and walked freely from one side to another side of the walkway, while the natural gait of the mice was recorded. The stride length, step length, front-rear print length, swing phase, and shortened foot-ground phase of changes in the rats were analyzed using the corresponding software.

**Histological Analysis**

For each group, the paws of mice were selected, sectioned, and stained for histopathological analysis, including H&E staining, safranin O-fast green staining, and immunofluorescence staining at the end of the treatment process to evaluate the therapeutic effect.

**Transcriptomic Analysis Methods**

Differential gene expression analysis was performed on the raw count data using the R package (limma). The adjusted *p*-value (*p* adj) < 0.05 and an absolute log2 fold change (LogFc) > 1 were considered statistically significant and defined as differentially expressed genes (DEGs) for GSE242872 (https://www.ncbi.nlm.nih.gov /geo/query/acc.cgi?acc=GSE242872) of Gene Expression Omnibus database (GEO) and our RNA subsequent analysis. To visualize the expression patterns of the DEGs across samples, a heatmap was generated using the pheatmap R package (pheatmap). Z-score normalized the expression values for each row to better illustrate variations among genes. Protein-protein interaction (PPI) networks for the DEGs were retrieved from the STRING database, and the resulting interaction data were imported into Cytoscape for further network visualization and analysis. For comparisons involving multiple groups, Venn diagrams were plotted using the R package (VennDiagram) to identify unique and shared DEGs among different experimental conditions. To elucidate the biological functions and pathways enriched in the DEGs, Kyoto Encyclopedia of Genes and Genomes (KEGG) pathway enrichment analysis was conducted using the R package (clusterProfiler). Terms with a corrected *p*-value < 0.05 were considered statistically significant.

**Supporting Figures**

**Figure S1 The stability and PDI values of TPR and TPR-LYTAC.**

**A and B.** The stability of TPR and TPR-LYTAC in various solutions (water, PBS, FBS, DMEM, and DMEM+FBS). **C and D.** PDI values of TPR and TPR-LYTAC in water.

**Figure S2** Quantitative analyses of relative fluorescence intensity in Figure 3H. Mean±SD, n = 3, ** *p* < 0.01, **** *p* < 0.0001.

**Figure S3** **A.** Cell viability of HUVECs treated with TPR and TPR-LYTACs at different concentrations for 24 h. **B.** Quantitative analyses of relative fluorescence intensity in Figure 4H. Mean±SD, n = 3, **** *p* < 0.0001, ns *p* ≥ 0.05.

**Figure S4** **A.** Surface MSR1 undergoes recycling throughout the time-dependent in TPR-LYTACs degradation process by flow cytometry testing. **B.** Quantitative analyses of relative fluorescence intensity in Figure 6E and 6G.

**Figure S5** **A.** Quantitative analyses of relative fluorescence intensity in Figure 7D. **B.** Quantitative analyses of relative fluorescence intensity in Figure 7F. **C.** Quantitative analyses of relative fluorescence intensity in Figure 7I. Mean±SD, n = 3, * *p* < 0.05, ** *p* < 0.01, *** *p* < 0.001, **** *p* < 0.0001.

**Figure S6** **A.** The NIR-II FL imaging of major organs and joints. **B.** The NIR-II FL ratio of joints/Liver. Mean±SD, n = 3, *** *p* < 0.001.

**Figure S7** Quantitative analyses of relative fluorescence intensity in Figure 9E. Mean±SD, n = 3, * *p* < 0.05, ** *p* < 0.01, *** *p* < 0.001, **** *p* < 0.0001, ns *p* ≥ 0.05.

**Supporting Video**

**Video S1.** The video captures the time-lapse imaging of M1 macrophages internalizing TPR-LYTACs-FITC and its subsequent colocalization with lysosomes. Signals are shown as green fluorescence for TPR-LYTACs-FITC, red fluorescence for lysosomes (Lyso-Tracker Red), and blue fluorescence for nuclei (Hoechst 33342), respectively.
